# A Cell Pre‐Wrapping Seeding Technique for Hydrogel‐Based Tubular Organ‐On‐A‐Chip

**DOI:** 10.1002/advs.202400970

**Published:** 2024-06-13

**Authors:** Jing Nie, Sha Lou, Andreas M. A. O. Pollet, Manon van Vegchel, Carlijn V. C. Bouten, Jaap M. J. den Toonder

**Affiliations:** ^1^ Microsystems Research Section Department of Mechanical Engineering Eindhoven University of Technology Eindhoven 5600 MB The Netherlands; ^2^ Institute for Complex Molecular Systems (ICMS) Eindhoven University of Technology Eindhoven 5600 MB The Netherlands; ^3^ Soft Tissue Engineering & Mechanobiology Research Section Department of Biomedical Engineering Eindhoven University of Technology Eindhoven 5600 MB The Netherlands

**Keywords:** cell pre‐wrapping seeding, hydrogel, renal proximal tubule, sacrificial template, tubular organ‐on‐a‐chip

## Abstract

Organ‐on‐a‐chip (OOC) models based on microfluidic technology are increasingly used to obtain mechanistic insight into (patho)physiological processes in humans, and they hold great promise for application in drug development and regenerative medicine. Despite significant progress in OOC development, several limitations of conventional microfluidic devices pose challenges. First, most microfluidic systems have rectangular cross sections and flat walls, and therefore tubular/ curved structures, like blood vessels and nephrons, are not well represented. Second, polymers used as base materials for microfluidic devices are much stiffer than in vivo extracellular matrix (ECM). Finally, in current cell seeding methods, challenges exist regarding precise control over cell seeding location, unreachable spaces due to flow resistances, and restricted dimensions/geometries. To address these limitations, an alternative cell seeding technique and a corresponding workflow is introduced to create circular cross‐sectioned tubular OOC models by pre‐wrapping cells around sacrificial fiber templates. As a proof of concept, a perfusable renal proximal tubule‐on‐a‐chip is demonstrated with a diameter as small as 50 µm, cellular tubular structures with branches and curvature, and a preliminary vascular‐renal tubule interaction model. The cell pre‐wrapping seeding technique promises to enable the construction of diverse physiological/pathological models, providing tubular OOC systems for mechanistic investigations and drug development.

## Graphical abstract

Targeting the bottleneck of cell seeding inside tubular channels with small dimensions and branching/curving geometries, a sacrificial template cell pre‐wrapping seeding technique is proposed to construct hydrogel‐based tubular organ‐on‐a‐chip models, contrasting the usual approach of injecting cells into pre‐constructed channels. The technique is demonstrated by a perfusable renal proximal tubule‐on‐a‐chip and cellular tubular structures with branches and curvature.

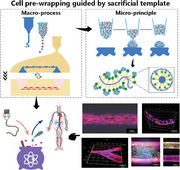

## 1 Introduction

The use of animal models has been the gold standard for pre‐clinical drug testing. However, the wide involvement of animal models is restricted due to ethical concerns, and interspecies differences are limiting their predictive performance.^[^ ^1^ ^]^ In vitro two‐dimensional (2D) cell culture, on the other hand, is far from representing the in vivo environment, since complicated geometrical, biochemical, and mechanical three‐dimensional (3D) environmental stimuli are missing.^[^ ^1^, ^2^ ^]^ Cells easily lose their key phenotypic and functional properties,^[^ ^3^ ^]^ which greatly limits the predictive value of current in vitro models for in vivo drug effects, as well as their capacity of simulating physiological/pathological mechanisms.^[^ ^2a^ ^]^ This highlights the demand for alternative representative models that can better mimic the structure and function of human tissues/organs. In the past decades, the development of organ‐on‐a‐chip (OOC) models has become a growing research field. Compared with animal or 2D models, such 3D microfluidic models can better recapitulate the cellular microenvironment in a controllable way.^[^ ^4^ ^]^ In particular, OOC models enable researchers to define and control the geometry and matrix material in which the cells are implemented. By closely mimicking the in vivo environment of cells, OOC models are expected to behave similarly to the in vivo tissue/organ, and thus the predictability for in vivo behavior will be improved.^[^ ^2^, ^5^ ^]^

However, current OOC models are limited by technical challenges caused by the inherent properties of the applied materials and the involved fabrication methods. In most microfluidic systems built through conventional fabrication methods such as photolithography, the channels have rectangular cross sections and flat walls. Hence, the geometry of tubular‐structured tissue/organs with circular cross sections, such as blood vessels, lymph vessels, and the nephron, is not represented well. This means that functional tissue properties that are directed by geometry, as well as flow‐induced cues such as shear stress, are not emulated well in conventional microfluidic systems. Also, polydimethylsiloxane (PDMS) is often used as microfluidic device base material,^[^ ^6^ ^]^ which is far too stiff to faithfully mimic the mechanical properties of in vivo extracellular matrix (ECM), nor are the morphological, chemical, and biological properties of ECM well represented by PDMS.^[^ ^1^, ^2^, ^7^ ^]^ Improved, but still limited geometries can be achieved based on the fiber retraction technique.^[^ ^8^ ^]^ The combination of filament extrusion‐based 3D printing technology^[^ ^2^, ^9^ ^]^ and sacrificial template molding^[^ ^2^, ^3^, ^10^ ^]^ offers possibilities for the construction of channels with circular cross section, curved walls, and flexible 3D geometries.^[^ ^11^ ^]^ The involvement of a wide range of natural and synthesized hydrogel materials makes it possible to simulate the in vivo matrix environment more closely.

Another limitation of current approaches to realizing OOC models is posed by cell seeding, which is a crucial step in the construction of any OOC model. A consistent and reproducible cell seeding method is demanded in order to achieve a uniform cell distribution as well as stable and controllable cell density.^[^ ^12^ ^]^ Conventionally, cells are seeded through microfluidic injection of a cell suspension into pre‐constructed channels.^[^ ^3^, ^5^, ^7^, ^8^ ^]^ Although this method is attractive and has good potential,^[^ ^9^ ^]^ there are several drawbacks. Direct and precise control over the cells is lacking, since only the injection parameters are adjustable. As a result, this cell seeding method makes it difficult to achieve homogeneous cell seeding and to reach a confluent cell monolayer over the entire channel walls. Due to the nature of the injection operation, there is a limit to the smallest channel dimension of ≈100 µm in which cells can be seeded, and the consistency between seeding results of different experimental groups cannot be guaranteed. For branching channels, an injected cell suspension preferentially flows into the branches with lower flow resistance, possibly leaving certain channels blank since cells do not reach them. Also, cells tend to settle and adhere preferentially to the bottom side of the channels due to gravity. As a result, elaborate operations are needed to realize coverage of the entire channel walls, such as flipping over or rotating the cell‐loaded constructs by specific angles after specific time intervals, or repeating the seeding step multiple times, which is time consuming and labor intensive.^[^ ^2a^ ^]^ Furthermore, injection cell seeding lacks full control over the exact number of cells seeded into the channels, since it is difficult to predict the number of cells that are actually attached as well as the number of cells being flushed out by further perfusion.

Different from the conventional OOC realization process that consists of microfluidic channel manufacturing and cell injection seeding, the development of 3D bioprinting technology has opened a new avenue for creating tubular‐structured OOC models. The development of suspension printing technology has improved the accuracy of extrusion‐based 3D bioprinting to a minimal microfilament diameter of 20 µm.^[^ ^13^ ^]^ The development of specially designed cell‐responsive bioinks has further advanced this technique.^[^ ^14^ ^]^ Among them, coaxial extrusion‐based printing enables to construct built‐in microchannels simultaneously.^[^ ^15^ ^]^ However, the major difficulty and limitation of this method lies in extending its application toward tubular OOC with interoperable bifurcations, such as present in the branches of blood vessels. In addition, it remains challenging to design a printing ink that meets the demands of both sufficient mechanical manufacturing strength and matrix biomimicking performance. Other methods harness cell self‐assembly to create a tubular model over time, such as obtained by the deformation of cell sheets into tubular structures,^[^ ^16^ ^]^ and the induction of cell proliferation and migration to form cellularized lumens. In combination with micro‐fiber extrusion‐based gel printing, researchers successfully built hydrogel fibers with a cell‐confluent monolayer at the border of the extruded fiber through cell migration.^[^ ^17^ ^]^ However, also this approach cannot directly realize branching structures, while the printing feasibility, precision, and flexibility are limited by the biological hydrogel ink. Also, these structures cannot be connected to an external microfluidic system due to the absence of a hollow lumen. Based on another approach that involves light‐assisted 3D printing, researchers have achieved precise cell distribution in pre‐defined patterns. However, the cell‐embedding 3D printing process puts limitations on the material selection and manufacturing conditions.^[^ ^18^ ^]^ As yet another approach, a pure biochemical strategy can be applied to create biomimicking lumens, such as the directional induction of angiogenesis from pre‐constructed large vessels by applying angiogenic signals or growth factors, such as present in blood capillaries,^[^ ^19^ ^]^ or by inducing vasculogenesis of blood vessel networks.^[^ ^20^ ^]^ However, this approach is limited to small constructs, since it is cost/time‐prohibitive. Also, the lack of a physical guidance hinders the realization of precise and pre‐defined cell and lumen patterns. Finally, the obtained models are often not compatible with external microfluidic perfusion system due to the challenge of realizing fluidic connections.

To address these limitations, we introduce an alternative cell seeding technique in which a carrier loaded with cells is pre‐wrapped around a 3D printed sacrificial sugar fiber template with a circular cross‐section. Printed by our previously developed 3D sugar printing technology,^[^ ^21^ ^]^ the fiber template can be tuned to have a diameter ranging from 50 µm to 1 mm. A hydrogel is subsequently cast around this construct and cured, after which the sugar fiber template is selectively dissolved. This results in tubular structures with a circular cross section, lined with a homogeneous and confluent monolayer of cells, within a hydrogel mimicking the properties of ECM. Finally, a microfluidic pump can be connected to the cellularized tubular lumens to apply perfusion, mimicking physiological mechanical and biochemical stimuli. The cell carrier involved in our cell pre‐wrapping seeding method acts as a temporal encapsulation for cells. It is extruded as single drop, which adheres directly onto the sacrificial template, while there is no need for it to hold a specific shape with mechanical strength or to form an integral construct. Also, printability and biomimicry are separated in our method, since printability is a demand put on the sacrificial carrier material, while the casted hydrogel must mimic ECM.

We demonstrate our method by creating a renal proximal tubule‐on‐a‐chip. The renal tubule is an essential part of the nephron, which is the basic functional unit of the kidney.^[^ ^22^ ^]^ However, it is challenging to mimic it because of its tubular structure, small diameter (40‐60 µm), and curved geometry. Most of the current models of the renal proximal tubule still lack key features of the in vivo nephron due to the limitations of current OOC models mentioned above, such as the small dimensions (diameter ≈50 µm),^[^ ^3^ ^]^ tubular geometry (circular cross section and 3D curvature),^[^ ^23^ ^]^ and soft matrix environment (≈4 kPa). We show that our method can realize these features. Also, a vascular system is usually not included in current models, which excludes the simulation of the main kidney functions since renal‐vascular interactions are key for these. As an initial demonstration of our method to create such an interaction model, we also built a preliminary vascular‐renal tubule coculture model based on this method.

Taken together, our approach will open up new possibilities to designing more realistic OOC models, the cell pre‐wrapping seeding technique opens an avenue to overcome limitations faced in traditional microfluidic cell seeding approaches, and it provides a universal and promising way to construct complex biomimicking cellularized lumens, particularly for the manufacturing of tubular OOC models with controllable shape and performance, it will promote the transition from still rather artificial microfluidic models to better emulation of living tubular systems, eventually contributing to a better understanding of human tissue/organ physiology and pathology.

## 2 Results

**Figure 1:**
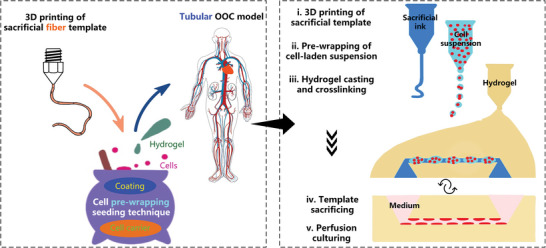
From 3D printed sugar fiber to tubular OOC models.

In combination with our previously developed 3D sugar printing technology,^[^ ^21^ ^]^ we propose an alternative cell seeding technique for the construction of tubular OOC models (**Figure** **1**). In short, the corresponding workflow is as follows: First, a sacrificial fiber template is fabricated through 3D sugar printing, after which the sugar fiber is double‐coated to protect it from early dissolution and for better cell adhesion. Next, cells are seeded by pre‐wrapping a carrier containing them around the coated sugar fiber. Then, hydrogel is cast around and cured. Finally, the whole construct is soaked in medium for sugar dissolution, creating a cellularized hollow lumen, and the model is connected to a perfusion system for dynamic culture.

### 2.1 3D sugar Printing of Fibers and Luminal Structures

**Figure 2:**
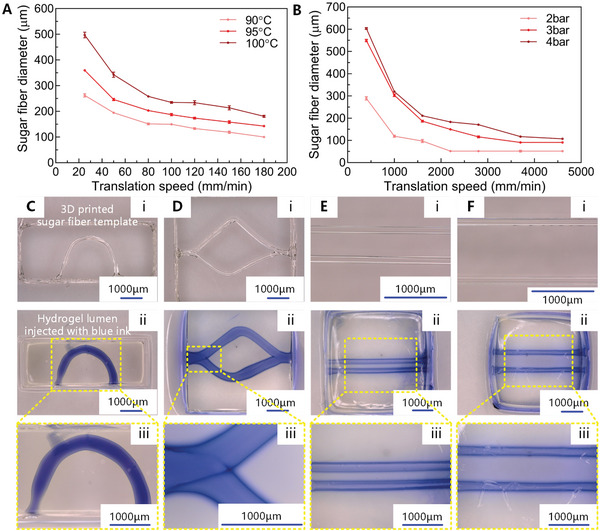
3D sugar printing to create luminal structures in hydrogel: effect of printing parameters on dimensions, and images of printed fiber and luminal geometries. A) Dependency of printed sugar (sucrose–glucose mixture) fiber diameter on nozzle translation speed and extrusion temperature at an extrusion pressure of 1 bar. B) Dependency of printed sugar (maltitol) fiber diameter on nozzle translation speed and extrusion pressure at an extrusion temperature of 135 °C. Images of printed sugar fiber structures with curved (Ci), bifurcating (Di), and parallel (Ei, Fi) geometries; the sugar fiber structures are suspended in a sugar frame at a height of 2 mm above the glass substrate. Perspective and detailed images of the GelMA casts around the fiber structures after sugar dissolution and injection of blue ink showing curved (Cii, Ciii), bifurcating (Dii, Diii), and parallel (Eii, Eiii, Fii, Fiii) luminal geometries.

As described in our earlier work, we can print suspended sugar fiber structures in‐air between supporting pillars or walls using our previously developed sugar 3D printer.^[^ ^21a^ ^]^ We systematically investigated the effects of the 3D printing parameters on the dimensions of the obtained sugar fibers. **Figure** **2**A,B shows that the diameter of the sugar fiber increases with increasing extrusion temperature and pressure, whereas it decreases with printing nozzle increasing translation speed. The effect of temperature can be explained by the fact that a higher extrusion temperature results in a lower viscosity of the molten sugar so that, at equal extrusion pressure, the quantity of sugar extruded per time increases, thus leading to an increase of fiber diameter. A higher extrusion pressure also leads to extruding more sugar, which again results in thicker fibers. The effect of nozzle translation speed happens because the fiber is stretched more at a higher speed and thus the fiber becomes thinner when the printing nozzle moves faster. The sugar fiber diameter can be adjusted ranging from 50 to 500 µm by regulating the extrusion temperature, the pressure, and the printing speed, for the sugar material systems consisting of a sucrose–glucose mixture and maltitol, respectively, as shown in Figure 2A,B. The data shown in Figure 2A,B demonstrate that our sugar printing method makes it possible to create sugar fibers in a controllable and reproducible way.

By prescribing the printing paths, a variety of tubular structures can be achieved. Figure 2Ci–Fi shows top‐view photos of the suspended printed sugar fiber structures, and Figure 2Cii–Fii shows images of gelatin methacrylate (GelMA) casts of the same structures after sugar dissolution and blue ink injection. Figure 2Ciii–Fiii shows detailed images of the ink‐injected GelMA casts. The sugar fiber structures are suspended in a sugar frame at a height of 2 mm above a glass substrate. The results show that it is possible to print sugar fibers of considerable length (15 mm) without the need for additional supports or junctions.

Figure 2C shows the feasibility of forming curved luminal structures in hydrogel, which allows for mimicking the geometry of a renal tubule. Figure 2D illustrates the possibility of forming a branched network similar to a bifurcating vascular system. Figure 2E,F displays examples of a parallel‐lumen structure with controllable distance (500–1000 µm) between the two lumens, which can be used as a coculture model to investigate the interaction between different tissues.

The 3D sugar printing process can be applied to fabricate sacrificial fibers for obtaining intricate networks of internal lumens with a wide range of dimensions and within multiple types of matrix materials. The flexibility and versatility of this technology enables a diversity of in vitro models to be built using multiple cell sources and matrix materials, providing the prospect of creating diverse OOC models for investigating physiological and pathological mechanisms, and, eventually, for drug testing.

### 2.2 Cell Pre‐Wrapping Seeding Technique

The most critical step of the whole workflow in our method is the loading of cells on the 3D printed sugar fiber structures. To achieve this, we developed a novel cell pre‐wrapping seeding technique. The concrete goal is to achieve an even cell loading with the final aim of forming a uniform and dense cell monolayer on the inner surface of a lumen with a circular cross section, within a hydrogel environment. In order to achieve this, two major issues must be solved: one is to encapsulate the cells and hold them in place, and the other is to place the cells uniformly on the outer surface of the printed sugar fiber.

**Figure 3:**
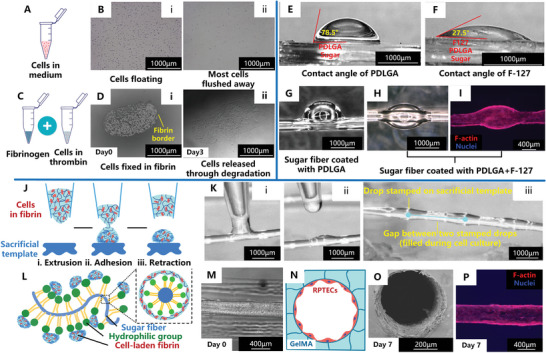
Key points of cell pre‐wrapping seeding. A) Schematic image of cells suspended in simple cell medium. B) Microscopy images of cells floating in cell medium (i), most of them being flushed away (ii). C) Schematic image presenting preparation of cell‐laden fibrin. D) Microscopy images of cells encapsulated in fibrin and fixed at an initial location (i), and released from fibrin through its degradation (ii). E) Microscopic display of the contact angle of a fibrin drop on the PDLGA coated sugar surface. F) Microscopic display of the contact angle of a fibrin drop on the F‐127 coated surface. G) Microscopic display of the fibrin droplets placed on PDLGA coated sugar fiber. H) Microscopic display of the fibrin droplets placed on F‐127 coated sugar fiber. I) Fluorescence micrograph of F‐actin/nuclei markers of the RPTEC monolayer formed through a cell‐carrying fibrin droplet placed on an F‐127 coated sugar fiber, after 7 days of culture. J) Schematic images illustrating the stamping seeding technique, including: i) extrusion of the cell‐laden fibrin solution; ii) adhesion of the cell suspension on the coated sugar fiber; iii) retraction of the remaining cell suspension. Ki,ii) Microscopic display of the stamping seeding process. Kiii) Microscopic display of the stamping seeding result. L) Schematic illustrations of sugar fiber bound with hydrophilic groups and stamped with cell‐laden fibrin, in laterial view, as well as cross‐section image showing the full wrapping of cell‐laden fibrin all around the fiber. M) Image showing the seeding result of RPTECs on sugar fiber with a diameter of 400 µm. N) Schematic image showing the cross section of the hydrogel lumen covered with epithelium. O) Microscopy morphology of the cross section of the RPTEC monolayer. P) Fluorescence micrograph of F‐actin/nuclei markers of the RPTEC monolayer formed by stamping seeding.

As shown in **Figure** **3**Bi, cells suspended in simple medium are in a free and floating state, and most cells are easily flushed away (Figure 3Bii). This is not compatible with the channel formation process, which happens after cell seeding.

The first issue is solved by using fibrin as a cell carrier to hold cells in place during the subsequent lumen formation process (hydrogel casting and curing, as well as sugar dissolution) based on its fast transition from liquid to gel once fibrinogen is mixed with thrombin.^[^ ^24^ ^]^ As shown in Figure 3C, the fibrin gel we use is composed of fibrinogen (10 mg mL^−1^) and thrombin (10 U mL^−1^) solutions at the ratio of 1:1 (v/v). Cells are suspended in thrombin solution at a pre‐calculated density (3.5e8 cells mL^−1^ for sugar fibers with a diameter of 50 µm), and further mixed with fibrinogen on ice. Then, the cell‐laden fibrin is immediately seeded on the sugar fiber. The volume of fibrin as well as the cell density should be tuned to the dimension of the sugar fiber to be seeded onto. In order to validate the feasibility of applying fibrin as a cell carrier, we characterized its cell embedding and fixing capability, its cell compatibility, and its degradation, as illustrated extensively in the Supporting Information text, and shown in Figure S1B (Supporting Information). Figure 3Di shows a clear border of the fibrin drop containing cells, which confirms that the fibrin allows for precise placement of the cells and can hold the cells at their initial seeding location. Furthermore, we confirm that the cells are able to degrade the fibrin within 24 h, and that they subsequently attach and spread over the substrate. The fibrin border gradually disappears and cells start to migrate on the surface, finally forming a cell monolayer, as shown in Figure 3Dii. This indicates that the fibrin is not present anymore, and cells are able to free themselves.

The second challenge is to load the cell‐laden carrier on the sugar fiber. Before cell seeding, the sugar fibers are coated with Poly (DL‐lactide‐co‐glycolide) (PDLGA), which is necessary to protect the fiber against premature dissolution.^[^ ^9^ ^]^ However, PDLGA is hydrophobic so that cell‐laden fibrin does not readily spread on it. The issue is solved by applying Pluronic F‐127 (F‐127) as a second, hydrophilic coating. Figure 3E,F shows that this second coating indeed decreases the contact angle of a fibrin droplet significantly. As a result, it improves the adhesion and spreading of the droplets placed on top. The droplets on the double‐coated fiber are more elongated and wrap around the whole fiber, as shown in Figure 3G,H. A dense cell monolayer is finally formed, as shown in Figure 3I.

To realize an even and smooth coverage of the cell‐laden fibrin on the sugar fiber surface, and finally achieve a confluent and uniform cell monolayer, we develop a “stamping” technique (other seeding techniques we attempted, with sub‐optimal results, are described extensively and in detail in the Supporting Information text, and the results are shown in Figure S5, Supporting Information). As shown in Figure 3J,K, cell‐laden fibrin is brought into contact with the double‐coated sugar fiber and then retracted. In this way, a small amount of residual fluid adheres to and wraps around the sugar fiber due to its hydrophilic surface, as schematically shown in Figure 3L.

For a sugar fiber with a length of 15 mm, a total volume of 6 µL cell‐laden fibrin is needed to achieve the complete stamping along the whole fiber. The small spacings between consecutive stamps (visible in Figure 3Kiii) are bridged and smoothened by subsequent cell proliferation and spreading during the following cell culture process. It is critical that quite a low volume of fluid is placed onto the fiber in this method. Therefore, in order to load a sufficient number of cells for forming a dense cell monolayer, a cell‐laden suspension with a relatively high cell concentration is required (3.5 times the cell concentration in the droplet‐placing seeding method, which is 3.5e8 cells mL^−1^ for a 50 µm lumen; the detailed calculation method of the cell density in fibrin is provided in the Supporting Information text).^[^ ^25^ ^]^ The cell seeding density for a 400 µm lumen is explored in the Supporting Information text, and shown in Figure S2 (Supporting Information). The stamping‐based cell pre‐wrapping seeding technique in combination with the fibrin cell carrier and the additional F‐127 coating leads to uniform cell loading along the sugar fiber (Figure 3M), finally resulting in a confluent cell monolayer all around the lumen and along the entire lumen length, as illustrated by Figure 3O,P. In order to obtain a full coverage, the stamping‐based cell deposition process involves the placement of multiple adjacent drops on the 3D printed fiber surface. In case of renal proximal tubule epithelial (RPTEC)‐ laden fibrin drops stamping‐placed on a PDLGA/F‐127 double‐coated sugar fiber, the adhered drop size is 1000 µm in diameter. On the one hand, in order to prevent the immediate aggregation or fusion between adjacent drops during the stamping process, the minimum center‐to‐center distance between two drops is 1500 µm. Correspondingly, there is a limitation to the minimal distance between two adjacent channels. In order to achieve independent pre‐loading of cells on separate adjacent templates, the channel distance and the cell‐suspension droplet size should be tuned to each other, depending on the properties of the selected cell carrier and the involved cell type. To avoid merging, the distance between adjacent cannels should be smaller than the sum of the drop height and the minimal gap between adjacent drops. For the material system applied in this manuscript (RPTECs in fibrin, PDLGA+F‐127 coating), the height of the drops placed on template is 43 µm, and the minimal gap between two adjacent drops is 500 µm, therefore the minimum distance between two adjacent template fibers is 543 µm. On the other hand, to ensure the later connection between cells in adjacent drops and the formation of a confluent/dense cell monolayer during culturing, the maximum center‐to‐center distance between two drops is 2000 µm. Compared with the conventional microfluidic injection cell seeding approach, this stamping‐based cell pre‐wrapping seeding technique omits the flipping or rotating steps normally applied to achieve uniform cell coverage, saving operation and waiting time.

### 2.3 Complete Workflow, Demonstrated by the Construction of a Renal Proximal Tubule‐On‐A‐Chip

**Figure 4:**
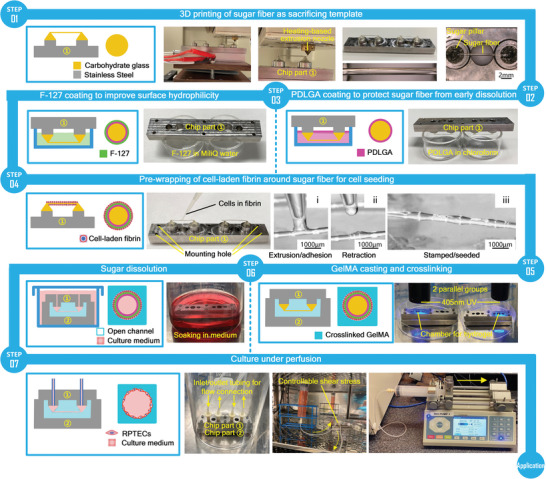
Complete workflow for engineering perfusable microfluidic tubular OOC models by pre‐wrapping seeding cells around 3D printed sacrificial sugar templates, applied to creating a renal proximal tubule‐on‐a‐chip. 1) 3D printing of a suspended sugar fiber structure as a sacrificial template. 2) PDLGA coating to protect the sugar fibers from early dissolution. 3) F‐127 coating to improve surface hydrophilicity for uniform cell seeding. 4) Pre‐wrapping seeding of cell‐laden fibrin around the sugar fiber; the cells are RPTECs for emulating the renal proximal tubule. 5) GelMA casting around the sugar fiber structure and subsequent UV crosslinking. 6) Sugar dissolution in cell medium. 7) Culture under perfusion after connection with a pump system.

**Figure** **4** presents the complete process of engineering a 3D microfluidic tubular microphysiological cellular model within a hydrogel and connected to a perfusion system, in this case representing a renal proximal tubule‐on‐a‐chip. The whole process consists of seven steps:

1. Fabricating a sacrificial fiber template by 3D printing of sugar materials, providing precise control over the dimensions and geometry of the fiber and corresponding tubular structures, as described in the previous section. The fiber is printed on tapered sugar pillars previously printed on a stainless steel holder on top of in‐ and outlets for eventual connection to a perfusion system.
2. Coating with PDLGA to protect the template from dissolving prematurely. The applied dip coating procedure is extensively described and schematically shown in Figure S3 (Supporting Information), and Ethyl cellulose (EC) coating is explored as another option in Supporting Information text. The thickness of the PDLGA coating is an important aspect. A discussion of the requirements of the coating and its effect on the final performance of the developed model can be found in the Supporting Information text and Figure S4 (Supporting Information).
3. Coating with F‐127 to improve hydrophilicity to facilitate sufficient and uniform cell seeding on the template surface, as described above. Polyvinyl alcohol (PVA) coating is explored as another option in Supporting Information text.
4. Pre‐wrapping seeding of cells in a fibrin carrier uniformly on the entire surface of the fiber template by the stamping‐based method described above. For the renal proximal tubule‐on‐chip, renal proximal tubule epithelial cells (RPTECs) were applied.
5. Casting hydrogel around the template and ultraviolet (UV) crosslinking the gel. We adopted GelMA as the matrix due to the fact that this material, synthesized based on natural gelatin, is easily cured in a reproducible way, with good mechanical properties after curing as well as excellent biological performance. 5% GelMA was chosen to represent the ECM, mimicking the stiffness of the in vivo renal microenvironment.^[^ ^26^ ^]^ The type and composition of the hydrogel can be adjusted to mimic the matrix properties of specific application scenarios. The biocompatibility of GelMA with RPTECs is validated in Supporting Information text, and shown in Figure S1A (Supporting Information).
6. Creating an inner luminal structure within hydrogel by soaking the construct in cell medium and dissolving the sugar template.
7. Forming a cell monolayer by culturing under low‐shear stress perfusion in connection with a pump system.

In order to recapitulate the properties of the native renal tubule and to establish suitable conditions for constructing the renal proximal tubule model, a series of parameters involved in the multiple steps of the whole workflow were systematically studied, including the sugar printing parameters and corresponding Gcodes, coating solution concentration, coating time, coating application method, cell pre‐wrapping strategy, cell density, as well as hydrogel concentration and crosslinking conditions. The optimal experimental conditions found during the course of the protocol development and modification are described extensively and in detail in Supporting Information text, and lead to the creation of a model of the renal proximal renal tubule. The detailed description of all steps, given in the Supporting Information text, also explicitly reports all the critical points in these steps. Troubleshooting advice can be found in Table S1 (Supporting Information). The approaches to applying coatings, as well as potential coating materials in order to achieve a protective and hydrophilic layer upon sugar fiber templates are explored in the Supporting Information text, and are shown in Figure S3 (Supporting Information).

### 2.4 Characterization of the Renal Proximal Tubule‐On‐A‐Chip

**Figure 5:**
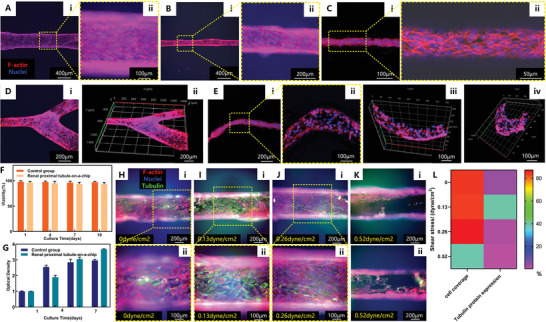
Characterization of the renal proximal tubule‐on‐a‐chip model. Fluorescence micrographs of the renal proximal tubule epithelium inside lumens with a diameter of 400 µm (A), 200 µm (B), and 50 µm (C) after 7 days of culture showing of F‐actin and nuclei markers. The cells are RPTECs and the matrix is GelMA. D) Fluorescence micrographs of the renal proximal tubule epithelium inside lumens with branching structures. E) Fluorescence micrographs of the renal proximal tubule epithelium inside lumens with curved shapes. F) Viability characterization of the RPTECs in the renal proximal tubule‐on‐a‐chip model compared to a control group. G) Proliferation characterization of the RPTECs in the renal proximal tubule‐on‐a‐chip model compared to a control group. H–K) Fluorescence micrographs (different magnifications) of the renal proximal tubule epithelium along a 400 µm lumen showing α‐tubulin, F‐actin, and nuclei markers, after 96 h of culture under different flow conditions: H) no flow (static), I) FSS = 0.13 dyne cm^−2^(physiological flow), J) FSS = 0.26 dyne cm^−2^, K) FSS = 0.52 dyne cm^−2^. L) Overall comparison of cell covered area and tubulin protein expression after 96 h of culture under different flow conditions.

We used optical microscopy to characterize the renal proximal tubule‐on‐a‐chip created by the cell pre‐wrapping seeding technique in combination with 3D sugar printing technology. The results are shown in **Figure** **5**.

A uniform and dense epithelial cell monolayer is formed, covering the entire length of the 15 mm long lumen (the epithelialization process of the lumen is described in the Supporting Information text, and is shown in Figure S6, Supporting Information). The morphology of the RPTECs within the lumens with diameters of 400 and 200 µm was visualized after 7 days culture by cell cytoskeleton (F‐actin) and nucleus DAPI staining, as displayed in Figure 5A,B for different magnifications. The individual channels of F‐actin and nuclei are displayed in Figure S8 (Supporting Information). The results confirm the feasibility of the proposed method to construct renal tubule models at different scales. It can be observed that RPTECs form a confluent monolayer inside the tubular channel and maintain their normal cellular morphology, with normal expression of F‐actin and nuclei. The cells present a healthy state, indicating the excellent biocompatibility of the proposed system.

Furthermore, we achieved to create a renal proximal tubule model with physiological dimension (diameter ≈50 µm), as shown in Figure 5C. Biomimicking branching networks and curved tubules of RPTECs were also constructed, as shown in Figure 5D,E. Such small and complex structures are difficult or impossible to create using conventional microfluidic injection cell seeding approaches.

Cell viability in the renal proximal tubule‐on‐a‐chip model was analyzed on days 1, 4, 7, and 10, and the results were compared with those of a control group. Figure 5F shows high viabilities of >90% for all culture times. Cell proliferation in the model was analyzed using the cell counting kit‐8 (CCK‐8) on days 1, 4, 7, and 10, and the results were compared with those of a control group. As shown in Figure 5G, the optical densities of the samples show a gradually rising trend with culture time, indicating a continuous increase of cell numbers, confirming our earlier observation based on the microscopy images. These results demonstrate the biocompatibility of our system, including all the involved materials and processes. The cytotoxicity of the multiple materials involved in the workflow is characterized in the Supporting Information text and the results shown in Figure S7 (Supporting Information) suggest low cell toxicity for all materials (including the sugar material, the PDLGA coating material, and the F‐127 coating solution). For viability, proliferation, and cytotoxicity analyses, control groups were cultured with full medium in a well plate without any additional substances. The RPTECs‐loaded GelMA constructs can be maintained for >1 month in cell culture medium at 37 °C in 5% CO_2_ before degradation.

Under physiological conditions, RPTECs are under constant fluid shear stress (FSS)^[^ ^1^, ^27^ ^]^ from continuous flow of pro‐urine in the renal tubule and they respond to changes in FSS by altering their morphology and protein expression patterns, which may affect the reabsorption and secretion process of the cells, essential for healthy renal functioning.^[^ ^28^ ^]^ α‐tubulin is a cytoskeletal protein and a major component of microtubules that plays a crucial role in the regulation of cell shape, intracellular transport, cell motility, cell migration, and cell division. Moreover, emerging evidence suggests that tubulin proteins may play a role in cellular stress response.^[^ ^29^ ^]^ To investigate the influence of FSS on the epithelium within the lumen, we applied controlled FSS to the constructs by perfusion with full medium at specific flow rates, including an FSS of 0.13 dyne cm^−2^ to mimic the physiological condition, as well as 2 and 4 times the in vivo FSS value. The flow was controlled by a syringe pump system for 96 h, and the results were compared with those of a static‐cultured group without applying FSS. All the experiments were performed at 37 °C in 5% CO_2_. The structure of the stainless steel chip for dynamic culture and the sugar frame structure for static culture are displayed in Figure S9 (Supporting Information). In comparison with static‐cultured groups shown in Figure 5H, RPTECs display a higher expression of α‐tubulin in the engineered model cultured under biomimicking‐FSS medium perfusion, as shown in Figure 5I. However, a higher FSS than present in vivo lowers the expression of α‐tubulin (Figure 5J), and even disrupts the integrity of cell monolayer within the lumen (Figure 5K). The individual channels of tubulin, F‐actin, and nuclei under different FSS are displayed in Figure S10 (Supporting Information). It can be clearly seen from the statistical results shown in Figure 5L that the epithelium cultured under physiological FSS condition expressed more tubulin protein compared with all the other groups, while the epithelium was disrupted under four times physiological FSS condition. These results underline the importance of creating the right flow conditions in the renal proximal tubule model. The ability to apply controlled perfusion in our system therefore not only helps provide nutrition supply for cell growth, but in contrast to static culture conditions, it also makes it possible to create physiological flow conditions necessary to correctly emulate in vivo functioning. Importantly, since our lumens have a circular cross section, the FSS applied to the cells is uniform along the entire epithelium; in conventional rectangular channels this is not the case since the FSS varies widely over the channel walls, which makes it impossible to create physiological flow conditions.

Together, these results indicate that our novel technique makes it possible to create renal proximal tubule‐on‐a‐chip models with different dimensions and various biomimicking geometries, displaying high RPTECs survival, and excellent attachment, proliferation, and uniform distribution of cells. The circular cross section of the lumens (with a controlled diameter between 50 and 400 µm) and the feasibility of controlled perfusion make it possible to mimic in vivo flow conditions, which is essential for obtaining a physiologically relevant model as indicated by the α‐tubulin expression levels of the cells.

### 2.5 Demonstration of a Preliminary Vascular‐Renal Tubule Coculture Model

By printing of parallel fibers spaced at a specific distance and pre‐seeding of different cells separately onto the different fibers, the utility of our technique can be extended to the construction of coculture models, which can be potentially applied in investigating the interaction between different tissues.

**Figure 6:**
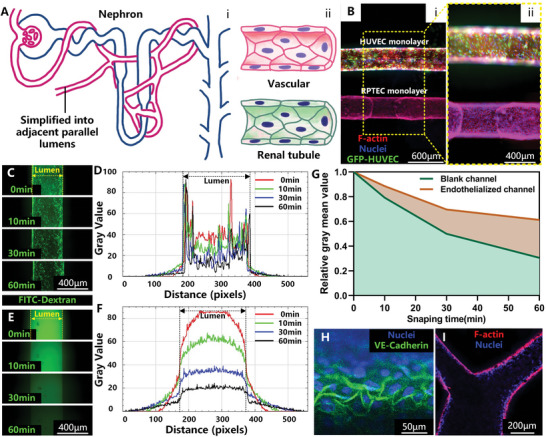
Demonstration of preliminary vascular‐renal tubule interaction model constructed by the cell pre‐wrapping seeding technique. Ai) Schematic of the nephron: the renal tubule is surrounded by vasculature. (Aii) Schematic diagram of the simplified adjacent parallel channel model. B) Fluorescence micrographs (different magnifications) of the engineered vascular‐renal tubule interaction model; HUVEC transfected with GFP: green, F‐actin: red, nuclei: blue. C) Time‐lapse confocal images of the permeating patterns of FITC‐dextran through the endothelialized lumen; HUVEC transfected with GFP: green. D) Profiles of the fluorescence intensity across the endothelialized lumen at different time points. E) Time‐lapse confocal images of the permeating patterns of FITC‐dextran through the blank lumen. F) Profiles of the fluorescence intensity across the blank lumen at different time points. G) Comparison of the permeability of FITC‐dextran through blank and endothelialized lumens. H) Fluorescence micrograph of nuclei (blue) and VE‐cadherin (green) markers of the endothelium. I) Fluorescence micrographs of the endothelium inside lumens with a branching structure.

Inspired by the in vivo nephron structure shown in **Figure** **6**Ai,^[^ ^7a^ ^]^ we designed and constructed a preliminary vascular‐ renal proximal renal tubule coculture model consisting of adjacent parallel endothelialized and epithelialized lumens as a proof of concept. To realize this model, we printed two parallel sugar fibers (400 µm diameter and 500 µm spacing) and pre‐wrapped seeding human umbilical vein endothelial cells (HUVECs) and RPTECs on the two respective fibers. This model is schematically displayed in Figure 6Aii. The microscopy images in Figure 6B show the morphologies of the green fluorescent protein (GFP)‐labeled endothelial cells and renal tubule epithelial cells (F‐actin marker). Both cells were observed to attach and spread well on the inner wall of the lumen, and indeed formed two parallel luminal structures separated by the GelMA matrix in between. These results demonstrate that our method has the potential to construct the renal tubular reabsorption and secretion unit through the realization of adjacent and parallel endothelial and renal tubular epithelial tubes. The individual channels of GFP, F‐actin, and nuclei in endothelium are displayed in Figure S11 (Supporting Information).

In order to initially evaluate the feasibility of applying this system in the simulation and investigation of the mass exchange and signal interaction between the renal filtrate and the blood, a very first analysis of the permeability of the endothelialized lumen was conducted in comparison with a blank lumen. We characterized the barrier function of the endothelium through the injection of fluorescein isothiocyanate (FITC)‐conjugated dextran (FITC‐dextran, 10 kDa) in the lumen, which simulates in vivo mass transport. It can be observed from the images captured at consecutive time points for the endothelized lumen and the blank lumen (Figure 6C,E, respectively) that the FITC‐dextran did permeate from the lumen into the hydrogel. The distribution of fluorescence intensity along the radial direction of the lumens at different time points is shown in Figure 6D,F for the endothelialized and the blank lumen, respectively. The distribution pattern exhibits a distinct profile, with singular peaks caused by the GFP‐transfected endothelial cells shown in Figure 6D. The permeability is obvious from the decrease of fluorescence intensity with time, as displayed in Figure 6G. The results show that the permeability of the empty lumen was significantly higher than that of the endothelialized lumen, proving the effective barrier function of the endothelium. Figure 6H displays the expression of vascular endothelial cadherin protein (VE‐cadherin) in the endothelial cell monolayer. It shows the formation of inter‐cellular junctions, which is critical for the integrity of the endothelium and the realization of barrier function. We also constructed a model of branching lumens lined with endothelial cells to showcase the potential of constructing biomimicking bifurcating vascular networks using this method, as shown in Figure 6I. In the future, we aim to extend this model to systematically studying the nephron filtration and reabsorption function, exploring kidney injury pathophysiology, as well as evaluating drug nephrotoxicity. In addition, a preliminary tumor‐vascular coculture model is illustrated in Supporting Information text, and displayed in Figure S15 (Supporting Information).

## 3 Discussion

The development of confluent cell monolayers inside perfusable channels within a matrix material is a fundamental prerequisite for the construction of a wide range of functional in vitro models^[^ ^11^, ^30^ ^]^ to be applied to physiological/ pathological studies and drug evaluation. In recent years, many studies have focused on the construction of microfluidic OOC platforms, and most of them are built through conventional fabrication approaches that result in in‐plane channels with rectangular cross sections, and that pose limitations to cell seeding. In the present study, we have proposed a novel technique for cell seeding inside microfluidic channels with circular cross sections, as well as a complete workflow for the construction of tubular‐structured OOC models. By combining the new cell pre‐wrapping seeding technique with the 3D sugar printing technology previously developed in our lab, we have demonstrated the feasibility of engineering a renal proximal tubule‐on‐a‐chip with a lumen diameter of 50 µm. We have also shown that we can create branched and curved luminal structures (the versatility of cell pre‐wrapping seeding is displayed in Figure S12, Supporting Information). Finally, we have shown the potential to construct a vascular‐ renal tubule coculture model. Such narrow and complex structures are very difficult or even impossible to achieve with conventional microfluidic injection cell seeding methods.

Using our sugar printer, we can fabricate intricate sacrificial templates with a wide range of dimensions and 3D geometries, onto which cells are seeded by the pre‐wrapping seeding method. The application of fibrin as a cell carrier during the cell pre‐wrapping‐seeding ensures that cells are deposited and fixed at the original seeding location to avoid being flushed away during further hydrogel casting and sugar dissolution processes, with full control over the number of cells seeded. The fast polymerization of fibrinogen once mixed with thrombin is favorable for the stability of the sugar fibers during seeding. Fibrin is known to degrade through cell‐associated enzymatic activities. We confirmed that fibrin can be degraded by the cells in one day given sufficient cell density (≥1e6 cells mL^−1^ in fibrin), which is favorable for cell proliferation and spreading along the whole lumen. After cell seeding, a hydrogel prepolymer solution is cast around the cell‐seeded structure and subsequently cured to form a 3D construct. The sugar fiber templates are easily removed through immersion in water or cell culture medium, creating a template‐shaped open luminal structure within the hydrogel. Almost all other existing methods can only create in‐plane structures. Our 3D sugar printing technology makes it possible to print suspended sugar fibers “in air” and control the fiber geometry in all three dimensions, as well as to realize branching and curved lumens. A truly 3D network can be created by printing fibers running over or underneath each other.^[^ ^21a^ ^]^ Moreover, the geometrical complexity of the model can be further enhanced by the programming of the 3D sugar printing code, resulting in biomimicking models of the native tissue/organ. The channel size and the distance between channels can be flexibly adjusted so as to apply multiple physical/biochemical simulations and to investigate the influence of geometry and flow conditions in specific aspects.

However, there are also some inherent limitations when applying 3D sugar printing technology in fabricating the sacrificial templates. In case of freestanding structures, there is an inevitable dragging and moving of the suspending sugar structures along with the nozzle during the further printing, which restricts the precision of the sugar structure and needs further compensation. Due to the inherent properties of the sugar, when exposed to air, its properties can be altered (especially in humid air). Therefore, the sugar material to be printed as well as the printed sugar structures have to be placed in a vacuum environment for long‐term storage. In order to perform the cell seeding and the hydrogel casting process, a hydrophobic coating is necessary to prevent early dissolution or deformation of the printed structures and to ensure the formation of circular cross‐sectional lumens with a constant diameter. In this study, PDLGA was chosen as a protective coating. The current coating solution formula and coating application operation need further optimization to reach a uniform coating layer as thin as possible while still providing sufficient protection for the sugar fibers. Furthermore, the degradation of the coating layer takes a long time,^[^ ^31^ ^]^ which might hinder the nutrients from reaching the cells within the lumen to a certain extent. Due to the involvement of multiple steps, this is a tedious and experimentalist‐depending technology at the current stage. In the current protocol, to realize the distribution of cells all over the whole fiber, cumbersome coating and multiple delicate stamping operations are needed, which are conducted by hand, and the stamping step is now extremely dependent on operator skills. Hand operation is labor‐intensive, and it lacks stability, controllability, and reproducibility, prohibiting scalable implementation, and restricting the size, precision, and complexity of the structures. Based on the fact of inevitable merging between adjacent drops and the limitation of minimal interval between two drops, for very closely‐situated channels, there might be the issue that a drop stamped aiming for one of the two adjacent fiber templates, will end up adhering to both fibers and connect the two channels in the end after template sacrificing. This issue may be further exacerbated by hand operation. Also, the mechanical stiffness of the GelMA hydrogel used is limited by the synthesis substitution degree and preparation concentration. New variants of GelMA or other hydrogel systems compatible with the proposed technique targeting at specific corresponding applications can also be considered. In addition to these technical limitations, there are also some biological limitations, such as suitability between fiber/ lumen size versus cell size, as well as the applicability to specific cells that are not eager to wrap or sit in a tubular environment.

Targeting these limitations, we will work on corresponding modifications of the whole workflow in future work. We will further enhance the 3D‐printing capabilities, by improvements of the sacrificial material composition and the printing characteristics. The optimization of sacrificial ink relates to its printability, strength, and self‐supporting capability, while ensuring its biocompatibility. The optimization of the printing process may include refining the coupling scheme between heating, extrusion, and cooling, optimizing the extrusion pressure profile, temperature, printing speed and other extrusion‐based 3D printing process parameters. This will enable to print more complex structures, potentially a complete nephron eventually. To enable the printing of very closely spaced channels, the accuracy can be improved by decreasing the height of deposited cell‐laden drop through the modification of the hydrophilicity of the template, the extrusion precision of the cell suspension, introducing a proper waiting interval between cell pre‐wrapping on adjacent fibers, as well as further optimization of the interaction between the cell‐laden suspension and the template surface.

Several steps (such as the double coatings and the stamping) can be potentially simplified or removed from the workflow through the combined modification of materials and processing settings, as well as the development and use of specially‐designed equipment. Specifically, the most sensitive step in the protocol is the stamping operation, which could in the future be conducted through automatic cell printing equipment with precise control over extrusion volume and timing, as well as accurate motion control. Through coupling nozzle extrusion and movement, it is feasible to realize accurate placement of cell‐laden drops at pre‐defined locations. The interaction force between cell suspension and sacrificial template can be accurately regulated to ensure that the structure of the sacrificial template will not be deformed or damaged.

Furthermore, although our system is proposed for and validated based on tubular OOC models that have a cell monolayer inside a lumen, it is also possible to extend the methodology to cells/tissues in other states, such as 3D cell aggregates, as displayed in Figure S13 (Supporting Information). The procedure will remain the same, but the protocol will require adaptations of the selected cell carrier, the cell loading operation, as well as the design of the template geometry. In addition to cell pre‐wrapping around the sacrificial template, the system could also be combined with other cell seeding techniques, leading to the presence of cells in different states at different locations, as demonstrated in detail in the Supporting Information text and schematically displayed in Figure S14 (Supporting Information).

In addition to implementing potential modifications of the method and the specific realization processes, our future research will involve more extensive and quantitative validation of the biological functions of the developed models of the renal tubule (e.g., permeability to different molecules), vasculature (e.g., nitric oxide production and endothelin secretion), as well as the interaction between them (e.g., reabsorption of albumin and glucose).

## 4 Conclusion

To summarize, we have presented a versatile strategy for the construction of tubular OOC models through the combination of a novel cell pre‐wrapping seeding technique with 3D sugar printing. Although some further modifications of this method are still needed to reach a robust workflow for general use, our protocol provides a promising basis for the construction of tubular tissue/organ models that can be integrated with multiple biochemical/physical factors to explore a variety of patho/ physiological phenomena, with potential applicability in regenerative medicine and drug development that demand various levels of biological complexity. Next to the concrete cases shown in this paper, the proposed workflow can further be adapted to facilitate the engineering of a wide range of other meaningful biomedical models, as illustrated in Figure S16 (Supporting Information). As such, the application of the cell pre‐wrapping seeding method can contribute to a better understanding of human tissue physiology and pathology, and may in the future be further developed and applied as a preclinical tool for evaluating and screening potential therapies.

## 5 Experimental Section

### Preparation of Sugar Material

The sugar material for printing was prepared by dissolving 53 g of sucrose (S9378, CAS Number: 57‐50‐1, Merck Life Science NV, Amsterdam, NL), 25 g of D‐(+)‐Glucose (G8270, CAS Number:50‐99‐7, Merck Life Science NV, Amsterdam, NL), and 10 g of dextran with an average molecular weight of 48–90 kg mol^−1^ (D3759, Merck Life Science NV, Amsterdam, NL) in 100 mL water (MilliQ, Darmstadt, DE) completely. The dissolving process took place on a stirring hot plate with an external thermometer (ETS‐D5, IKA RH, Staufen, Germany) by heating to 149 °C until all water was evaporated. The prepared sugar material was poured into barrels preheated to 120 °C with nozzles already fitted in. The prepared sugar material was stored in a vacuum desiccator (VWR International BV, Amsterdam, NL) for further use.

### 3D Printing of Sugar Fiber Template

The model was designed and the corresponding Gcode was edited through Repetier‐Host software. A pressure‐based 3D printer developed by the lab was used to print the sugar structures. The prepared sugar material was used as the printing material. Before printing, the top part of the stainless steel chip (printing substrate) was placed on the printing platform and fixed using a vacuum chuck. After that, an initialization process was run to define the position of the printing start point. The sugar material was heated to 90 °C, and the molten sugar was extruded by compressed air and deposited following the designed printing path which is controlled by G‐code. The printing time for each structure depended on the dimension and geometry of the sugar structure. The printed sugar structures were stored in a vacuum desiccator until further use.

### Protective Coating

Poly(D,L‐lactide‐co‐glycolide) (PDLGA) beads (P1941, Merck Life Science NV, Amsterdam, NL) were weighed and added to chloroform (288 306, CAS Number: 67‐66‐3, Merck Life Science NV, Amsterdam, NL) in a concentration of 12.5 mg mL^−1^. The mixture was placed at −20 °C overnight before ready to use. 5 ml PDLGA solution was added into a glass petri dish. The top part of the stainless steel chip with printed sugar structures was flipped over and placed on top of the glass petri dish with all sugar parts submerged into the PDLGA solution. The structure was lifted out of the PDLGA solution after 5 min, and left in fumehood for 10 min to evaporate the residual chloroform.

### Hydrophilic Coating

Pluronic F‐127 (F‐127) powder (P2443, CAS Number: 9003‐11‐6, Merck Life Science NV, Amsterdam, NL) was weighed and added to MilliQ water in a concentration of 10 mg mL^−1^. The mixing was finished by vortexing for 10 min. Five milliters of F‐127 solution was added into a glass petri dish. The top part of the stainless steel chip with PDLGA‐coated printed sugar structures was flipped over and placed on top of the glass petri dish with all sugar parts submerged into the F‐127 solution. The structure was lifted out of the F‐127 solution after 1 min, and immediately dried using a nitrogen gun. The double‐coated sugar structure was stored at room temperature in vacuum until further use.

### Cell Culture

RPTECs (RPTEC/TERT1, ATCC‐CRL‐4031, LGC Standards GmbH, Wesel, DE) were cultured in Dulbecco's modified Eagle's medium (DMEM): F12 medium (ATCC‐30‐2006, LGC Standards GmbH, Wesel, DE) supplemented with hTERT Immortalized RPTEC Growth Kit (ATCC‐ACS‐4007, LGC Standards GmbH, Wesel, DE), 10% fetal bovine serum (FBS) (16 140 071, CAS Number: 64742‐49‐0, Fisher Scientific, Landsmeer, NL), 1% (v/v) Penicillin/Streptomycin (15 140 122, Fisher Scientific, Landsmeer, NL), and Geneticin (G418) (0.1 mg mL^−1^, 11 558 616, Fisher Scientific, Landsmeer, NL). GFP‐HUVECs were obtained through lenti‐viral transfection. The GFP construct was inserted, including a puromycin resistance for selection (pLKO.1‐puro GFP). GFP‐HUVECs were cultured in Endothelial Cell Growth Medium 2 (EGM‐2) (C‐22211, Bio‐Connect B.V., TE, NL) supplemented with Endothelial cell growth factor kit (C‐39211, Bio‐Connect B.V., TE, NL), 1% (v/v) Penicillin/Streptomycin, and puromycin (0.5 µg mL^−1^, 15 490 717, CAS Number: 53‐79‐2, Fisher Scientific, Landsmeer, NL). HUVECs were discarded after 8 passages to ensure the representation of key endothelial characteristics. MDA‐MB‐231 cells were cultured in RPMI 1640 Medium (Gibco 21 875 034, Fisher Scientific, Landsmeer, NL) supplemented with 10% FBS and 1% penicillin–streptomycin. All cells were incubated at 37 °C in 5% CO_2_ in polystyrene tissue culture flasks. They were fed with fresh medium every other day and passaed every 4 days.

### Preparation of Cell‐Laden Fibrin Solution

Fibrin was used as a carrier for the RPTECs and HUVECs. Thrombin from bovine plasma (T4648, CAS Number: 9002‐04‐4, Merck Life Science NV, Amsterdam, NL) was dissolved in sterile Phosphate‐Buffered Saline (PBS) (15 374 875, Fisher Scientific, Landsmeer, NL) in 100 U mL^−1^ stock concentration and storage at −20 °C. Before use, the stock thrombin was thawed at room temperature, diluted in cold full medium into concentration of 10 U mL^−1^, and filtered through a 0.22 µm filter (Minisart, sterile RC, 16–0907, VWR International BV, Amsterdam, NL). Fibrinogen from human plasma (≥ 80% protein clottable) (F8630, CAS Number: 9001‐32‐5, Merck Life Science NV, Amsterdam, NL) was dissolved in cold full medium in a concentration of 10 mg mL^−1^ and filtered through a 0.22 µm filter. The culture flask with 90% RPTECs confluence was washed with PBS and incubated with 0.25% Trypsin‐ethylene diamine tetraacetic acid (EDTA) (11 580 626, Fisher Scientific, Landsmeer, NL) for 5 min at 37 °C in 5% CO_2_ to detach the cells from the culture flasks. The cell suspension was centrifuged at 900 rpm for 5 min at room temperature. The culture flask with 90% HUVECs confluence was washed with PBS and incubated with Accutase solution (C‐41310, Bio‐Connect B.V., TE, NL) for 5 min at 37 °C in 5% CO_2_ to detach the cells from the culture flasks. The cell suspension was centrifuged at 900 rpm for 5 min at room temperature. For the preparation of cell‐laden fibrin solution for seeding, the calculated number of cells was suspended in a 10 U mL^−1^ thrombin solution, and then mixed with a 10 mg mL^−1^ fibrinogen solution at ratio of 1:1 on ice.

### Preparation, Casting, and Crosslinking of GelMA Prepolymer Solution

Photoinitiator lithium phenyl‐2,4,6‐trimehtylenzoylphosphinate (LAP) (900 889, CAS Number: 85073‐19‐4, Merck Life Science NV, Amsterdam, NL) was dissolved in blank DMEM‐F12 medium in concentration of 0.5% (w/v) and filtered to sterilize (Minisart, sterile RC, pore size 0.2 µm). GelMA prepolymer solution was prepared by dissolving the freeze‐dried PhotoGelMethacrylated Gelatin (GelMA) (degree of substitution 80%, 5208, CellSystems GmbH, Troisdorf, DE) in DMEM‐F12 medium containing LAP (0.5% w/v) at a water bath (37 °C) in a concentration of 5% (w/v). The GelMA prepolymer solution was filtered through a 220 nm filter until further use. The GelMA prepolymer solution was poured onto the sugar structure for casting, and the crosslinking of GelMA prepolymer was achieved by exposing the GelMA casts to a 405 nm UV light for a specific time depending on the construct volume and shape.

### Preparation of Cell‐Laden GelMA Prepolymer Solution

GelMA hydrogel was used as a carrier for the tumor cells. Culture flasks with 90% MDA‐MB‐231 confluence were washed with PBS and incubated with 0.25% Trypsin‐EDTA for 3 min at 37 °C in 5% CO_2_ to detach the cells from the culture flasks. Next, the cell suspension was centrifuged at 1000 rpm for 5 min at room temperature, the supernatant was discarded, and the cells were resuspended in the prepared GelMA prepolymer solution with a concentration of 2e6 cells mL^−1^.

### Cell Activity Analysis

The cell viability of the samples was tested by a cell LIVE/DEAD assay. First, the samples were washed with PBS three times before being stained. After that, the samples were stained using LIVE/DEAD assay reagents (FungaLight Yeast CFDA, AM/Propidium Iodide Vitality Kit, Invitrogen F34953, Fisher Scientific, Landsmeer, NL) according to the kit instructions. Calcein AM and propidium iodide (PI) were diluted with PBS at a concentration of 2 and 8 µm, respectively. After incubation with the Calcein AM/PI mixture for 30–45 min in dark, the samples were washed with PBS to remove residual reagents. Finally, a fluorescence microscope (DMi8, Leica Microsystems B.V., Amsterdam, NL) was used to image the samples by acquiring two images of each frame: green for live cells and red for dead cells, respectively. The cell viability was calculated as: (1‐ the number of red stained cells/the number of total cells) × 00%.

### Cell Proliferation Analysis

The cell numbers of the samples after 1, 4, 7, and 10 days of culture were tested using a cell counting kit‐8 (CCK‐8) (96 992, Merck Life Science NV, Amsterdam, NL). The WST‐8 in the kit can be redoxed by intracellular dehydrogenase while generating an orange‐yellow formazan dye, which can dissolve into medium. The amount of formazan is proportional to the number of living cells. According to the instruction, the samples to be tested were taken out of the device and placed in a 24‐well plate. Next, the samples were washed with PBS three times. Then, 500 µL of medium and 50 µL of CCK‐ 8 reagent were mixed and added to each well. Finally, after 3 h of incubation, the solution was transferred to a 96‐well plate, with 100 µL for each well in five parallel groups. The optical density at a wavelength of 450 nm was measured with a microplate reader (BioTek Synergy HTX Multimode Reader, Agilent Technologies Netherlands B.V., Amstelveen, NL).

### Cytotoxicity Testing

The cell numbers of the samples cultured with addition of materials involved in the fabrication process were tested using CCK‐8. The WST‐8 in the kit can be redoxed by intracellular dehydrogenase while generating an orange‐yellow formazan dye, which can dissolve into medium. The amount of formazan is proportional to the number of living cells. According to the instruction, RPTECs were seeded in 24‐well transwell plates at a density of 5e5 cells per well and incubated for 24 h for cell adherence. Next, the materials to be tested (sugar material, PDLGA coating film, F‐127 coating solution) were added to each well and cultured for 48 h. The control group was incubated without adding any additional materials. Then, the culture medium was removed and samples were washed with PBS three times, after which 500 µl of medium and 50 µl of CCK‐8 reagent were mixed and added to each well. Finally, after 3 h of incubation, the solution was transferred to a 96‐well plate, with 100 µl for each well in five parallel groups. The optical density at a wavelength of 450 nm was measured with a microplate reader.

### Cell Morphology Analysis

The morphology of the cells within samples was visualized by cell cytoskeleton staining, including F‐actin and nucleus staining. According to the kit instructions, F‐actin and nucleus staining was conducted using Invitrogen ActinRed 555 ReadyProbes Reagent (Rhodamine phalloidin) (15 119 325, Fisher Scientific, Landsmeer, NL) and Invitrogen NucBlue Fixed Cell Stain ReadyProbes Reagent (DAPI) (12 333 553, Fisher Scientific, Landsmeer, NL). First, samples were washed with PBS, and fixed with 4% paraformaldehyde for 30 min, after which they were washed with PBS again and permeabilized with 0.5% Triton X‐100 for 5 min. Then, the samples were washed with PBS again, and incubated with ActinRed 555 ReadyProbes Reagent (2 drops ml^−1^) for 1 h in dark. In the next step, they were washed with PBS again, and incubated with NucBlueTM Fixed Cell Stain ReadyProbeTM reagent (2 drops ml^−1^) for 30 min in dark. Finally, the samples were washed with PBS one more time and imaged using a fluorescence microscope.

### Scanning Electron Microscopy Analysis

The samples were sliced from radial direction for observing the cross section of the lumen and fixed with 4% paraformaldehyde (Solarbio Co., Ltd., Shanghai, China) for >4 h at room temperature and fixed with tannic acid for 2 h. The samples were then dehydrated in a series of ethanol solutions (30%, 50%, 70%, 80%, 90%, 95%, and 100%) by soaking the samples in each solution for 30 min. Subsequently, the samples were dried at critical point. Finally, the constructs were coated with platinum in a sputter coater (Ion Sputter E‐1045, Hitachi, Tokyo, Japan), and imaged by the SEM system (SU‐6600, Hitachi, Tokyo, Japan).

### Immunostaining of Epithelial Monolayer

The epithelium formed under shear stress as well as static conditions were immunostained using tubulin antibody to investigate the influence of shear stress on the expression of cilia. For immunostaining, the samples were fixed in 4% paraformaldehyde for 30 min and permeabilized in 0.2% Triton X‐100 for 5 min. After that, the samples were blocked in 0.01% Triton X‐100 containing 10% horse serum for 1 h at room temperature and incubated overnight in tubulin primary antibody (Anti‐Acetylated Tubulin antibody, Mouse monoclonal, T7451, Merck Life Science NV, Amsterdam, NL) in 1:200 dilution. Then, the samples were incubated in 1:500 dilution of secondary antibody (Alexa Fluor 488‐conjugated Goat anti‐Mouse IgG2b Cross‐Adsorbed Secondary Antibody, 10 337 122, Fisher Scientific, Landsmeer, NL) for 1 h at room temperature. In the next step, samples were incubated in ActinRed 555 ReadyProbes Reagent and NucBlueTM Fixed Cell Stain ReadyProbeTM reagent for 1 h and 30 min, respectively. Finally, the samples were imaged using a fluorescence microscope.

### Barrier Function of the Endothelialized Lumen

In order to assess the permeability of the endothelialized lumen and the barrier function of the endothelium inside the lumen, culture medium was removed from the lumen, and the permeability was quantified by perfusion of culture media containing 25 µg mL^−1^ FITC‐conjugated dextran (FITC‐dextran, 10 kDa, TCI). As a comparison, empty lumens without endothelial cells seeded on it were constructed and injected with the prepared dextran solution. The diffusion patterns of FITC‐dextran were detected and captured using a fluorescence microscope. The fluorescent images at 0, 10, 30, and 60 min were extracted and the fluorescent intensity was quantified using ImageJ software.

### Immunostaining of the Endothelial Cells

After 7 days of culture, VE‐cadherin antibody (Santa Cruz Biotechnology) immunostaining was performed on the hydrogel constructs to investigate the inter‐cellular connection of the endothelial cells. For immunostaining, the cells were fixed in 4% paraformaldehyde or anhydrous methanol for 30 min and soaked in 0.5% Triton X‐100 for 30 min to permeabilize the cell membrane. Upon permeabilization, the samples were blocked in PBS containing 5% bovine serum albumin (BSA, Sigma) for 1 h at room temperature and incubated in VE‐cadherin primary antibody in 1:200 dilution overnight. Then, the hydrogels were incubated in 1/500 dilution of Alexa Fluor 488‐conjugated donkey anti‐rabbit secondary antibody (Invitrogen) for 2–3 h at room temperature (25 °C). In the next step, samples were incubated with DAPI for 30 min. Finally, the samples were imaged by a fluorescence microscope.

### Statistical Analysis

Data are expressed as mean ± standard deviation of independent replicates, N = 5 per time point and per condition group. Statistical analysis was conducted using ANOVA, and statistical significance was determined at P<0.05, as implemented in GraphPad Prism 9.5.1.

## Conflict of Interest

The authors declare no conflict of interest.

## Supporting information

Supporting Information

## Data Availability

The data that support the findings of this study are available from the corresponding author upon reasonable request.
