# Supplementary material for: A Cell Pre‐Wrapping Seeding Technique for Hydrogel‐Based Tubular Organ‐On‐A‐Chip
Source: Adv Sci (Weinh). 2024 Jun 13;11(30):2400970. doi: 10.1002/advs.202400970 (PMC11321624; doi:10.1002/advs.202400970)
Supplement: Supplementary file 1 — Supporting Information [file ADVS-11-2400970-s001.pdf]

ADVANCED SCIENCE
Open Access

# Supporting Information

for *Adv. Sci.*, DOI 10.1002/advs.202400970

A Cell Pre-Wrapping Seeding Technique for Hydrogel-Based Tubular Organ-On-A-Chip

*Jing Nie, Sha Lou, Andreas M. A. O. Pollet, Manon van Vegchel, Carlijn V. C. Bouten and Jaap M. J. den Toonder**

# Supporting Information

## A Cell Pre-wrapping Seeding Technique for Hydrogel-based Tubular Organ-on-a-chip

*Jing Nie, Sha Lou, Andreas M. A. O. Pollet, Manon van Vegchel, Carlijn V. C. Bouten, Jaap M. J. den Toonder**

**Step-by-step protocol**

1. Preparation of sugar material

Option 1: Sucrose–glucose mixture (Timing 2.5 h)

1) Empty the barrel of the sugar 3D printer filled with sugar material by continuous extrusion: insert barrel, heat the barrel to 120 °C, and place a boat underneath to collect the unwanted material; Another option is to clean the barrel by soaking it in 100 °C water, and change the water for 3-5 times.

2) Disassemble the barrel and nozzle, soak them in boiled water, and refresh the water after 5 min.

3) Take the barrel and nozzle out from the water, and dry them through compressed air blowing.

4) Assemble the barrel and nozzle using wrench, and place it on a steel rack.

5) Set oven temperature at 120 °C.

6) Place the rack with barrels in the oven.

7) Cover the hotplate with thin aluminum foil, and make sure air can still flow underneath for exchange of heating energy.

8) Place a bucket with cold water next to the setup for emergency.

9) Fill beaker with 100 mL MQ water, and place the beaker with stirring bar in the middle of the hotplate.

10) Insert the thermometer, make sure the neck part is immersed in liquid, and connect it to the hotplate.

11) Set hot plate temperature at 320 °C, stirring speed at 300 rpm, and thermometer temperature at 149 °C.

12) Check the stability of the setup: sensor bar fixed well, magnetic bar stirring smoothly and evenly.

13) Weigh 56 g Sucrose and 25 g Glucose, and weigh 10 g Dextran in a separate boat.

14) Add the materials to water, first dextran, followed by sucrose and glucose.

15) Heat up and stir on, turn off the heating to keep water temperature within 85-95 °C before all materials are dissolved.

16) Heat up to 149 °C after all materials are dissolved, and boil the sugar solution until all water is evaporated (no large bubbles left and in color of light brown)

17) During the process, keep an eye on the setup: temperature under control, sensor bar immersed and stable, magnetic bar stirring stably.

18) Retrieve the barrels from the oven and pour the prepared sugar material into them.

19) Make sure not to overfill, as the screw thread should be clean for further assembly

20) Cool down everything to room temperature

21) Store the barrel filled with sugar material in a vacuum desiccator with silica beads.

PAUSE POINT: the prepared sugar material should be stored in a vacuum desiccator to prevent hydration. Use of sugar materials that are older than 1 month is not recommended, because long-term storage may induce a change in the composition and printing properties of the sugar material, which might influence printing results.

CRITICAL STEP: Keep the temperature lower than 90 °C before all sugar is dissolved. The sensor bar should not touch the wall of beaker, the neck of sensor bar should always be immersed in liquid, and the magnetic bar should not touch sensor when rotating. Make sure all water is evaporated from the sugar material. At the same time, stop cooking as soon as all water is evaporated to avoid over-cooking.

Option 2: Maltitol (Timing 40 min)

Maltitol doesn’t need to be dissolved in water. Heat maltitol to 150 °C, and keep the temperature for 30 min until maltitol is fully melting every time when adding new maltitol.

2. 3D printing of sugar fiber as sacrificial template (Timing 2 h)

1) Design printing pathway based on the specific design of the sugar fiber template shape. Use Repetier-Host to program it into G-code. Set the barrel temperature and printing speed, and activated the nozzle.

2) Switch on the sugar printer, computer, and compressed air. Screw the cap on the barrel, place the barrel (with nozzle, filled with sugar material) inside the heating mantles and fix it tightly.

CRITICAL STEP: The heating mantles control the temperature of the barrels. The barrels are kept in place with a V-groove and set screw. Different nozzle sizes can be mounted to the barrels, to be able to print different diameters.

3) Open Repetier-Host software, connect machine with software, set temperature for extruder and bed, activate heating, wait until reaching sugar material melting point.

CRITICAL STEP: For printing of sucrose–glucose mixture, a pressure of 1 bar is applied unless stated otherwise. For printing of maltitol, a temperature of 135 °C is applied unless stated otherwise.

4) Place the top part of the stainless steel chip (printing substrate) on a glass slide, and keep it in place through a vacuum chuck.

5) Use manual control to finetune the position of the nozzle and printing platform to find the coordinates of the starting point of printing. Load initialization G-code, edit the G-code, copy the coordinates of the manual control into the initialization G-code. Run updated initialization process, set XYZ zero

CRITICAL STEP: Find Z origin using a piece of paper, a little friction indicates the reaching of the desired height.

6) Load the pillar printing G-code and run pillar printing process. Repeat initialization and pillar printing at another spot. Pillars with a height of 1.75 mm are printed on the inlet and outlet of the stainless steel chip.

CRITICAL STEP: Use manual control to raise the nozzle up a bit every time after the initialization process, in order to clean the nozzle and substrate before starting printing sugar structures. Use fiber-free tissue to clean nozzle.

7) Initialize the nozzle onto one of the pillars to define the starting point for fiber printing. Load and run the fiber printing G-code, and run fiber printing process. Print a free-standing fiber between the two pillars in form of either single fiber or network. Take the top part of the chip with printed structures from platform and store it in vacuum

PAUSE POINT: the printed sugar structures need to be stored under low humidity environment to preserve shape and structure.

3. PDLGA coating to protect sugar fiber (Timing 15 min)

1) Add chloroform in a glass bottle, add PDLGA into chloroform in concentration of 12.5 mg $ml^{-1}$ (w/v). Place the mixture at -20 °C for overnight, and the PDLGA solution is ready to use. 20 ml coating solution is sufficient to coat four sugar fibers on two stainless steel chips.

!CAUTION: Chloroform is toxic. Use personal protective equipment to avoid contact with skin and eyes. Open it only inside the chemical fumehood. Collect and discard waste appropriately.

CRITICAL STEP: Close the cap all the time to prevent chloroform evaporation. Chloroform cannot contact any plastic materials, e.g. container, pipette.

2) Add 5 ml PDLGA solution in a glass petri dish, flip over the top part of the stainless steel chip, and place it on top of the glass petri dish.

CRITICAL STEP: Make sure the sugar pillars and fibers are submerged into the PDLGA solution completely, while the stainless steel parts are not soaked in the solution. Multiple groups can be coated at the same time.

3) Wait for 5 min, lift up the sugar fiber out of PDLGA solution. Leave it in fumehood for 10min to evaporate extra chloroform.

CRITICAL STEP: Lift up the top part of the chip gently and slowly to make sure the PDLGA film formed on top of the solution is evenly wrapped around the sugar structures.

4. F-127 coating to improve surface hydrophilicity (Timing 5 min)

1) Add F-127 to MiliQ water in concentration of 10 mg $ml^{-1}$ (w/v). Use a glass rod or vortex to stir until F-127 is dissolved in MiliQ water completely, and the F-127 solution is ready to use. 20 ml coating solution is sufficient to coat four sugar fibers on two stainless steel chips.

2) Add 5 ml F-127 solution in a glass petri dish, flip over the top part of the stainless steel chip, and place it on top of the glass petri dish.

CRITICAL STEP: Make sure the sugar pillar and fiber parts are submerged in the F-127 solution completely, while the stainless steel parts are not soaked in the solution.

3) Wait for 1 min, and lift up the sugar fiber out of F-127 solution. Immediately use a nitrogen gun to dry the sugar structure completely.

CRITICAL STEP: Coat the sugar structure one-by-one in order to lift the sugar structure out from the F-127 solution and remove excess F-127 coating solution immediately to prevent sugar dissolution in the MilliQ water-based coating solution.

PAUSE POINT: The coated samples can be stored at room temperature in vacuum.

5. Pre-wrapping of cell-laden fibrin around sugar fiber for cell seeding (Timing 1 h)

1) Place the coated sugar fiber under UV for sterilization for 30 min.

CRITICALSTEP: From this step forward, all the steps should be performed inside the biological safety cabinet using aseptic technique to maintain the sterility of every part.

2) Weigh 0.01266 g fibrinogen in a vial, and add 1 ml cold medium into it. Pipette it for several times until fibrinogen is completely dissolved in medium. Filter the prepared 10 mg $ml^{-1}$ fibrinogen solution using 0.22 um sterilized RC filter, and make aliquots according to the number of samples to seed. Place all the aliquots on ice, and they are ready to use.

CRITICAL STEP: Use cold medium to dissolve fibrinogen and conduct all steps on ice to prevent solidification.

3) Dilute 100 U $mL^{-1}$ thrombin solution into 10 U $ml^{-1}$ using cold medium, and it is ready to use.

4) Prepare a cell-laden thrombin solution as follows: detach RPTECs using trypsin for 5 min at incubator. Neutralize the trypsin by adding 8mL cell medium and use the NucleoCounter® NC-200™ to count the cell concentration. Calculate the needed volume of cell suspension based on cell concentration and demanded cell number. Take the calculated volume of cell suspension in a separate vial. Centrifuge at 900rpm for 5 min. Discard the supernatant and remove the last bit using a 200 uL pipette. Re-suspend the cells in thrombin at a cell concentration of 3.5e7 cells $ml^{-1}$.

!CAUTION: The cells should be regularly checked to ensure that they are authentic and are not infected with mycoplasma.

CRITICAL STEP: Keep the incubation time accurate to avoid over-digestion of the cells. Check with a microscope whether all cells are rounded up and dettached. Tap/gently shake the flask to dislodge any remaining cells. Pipette to distribute cells uniformly in medium to get accurate cell concentration result. Avoid strong pipetting to prevent cell damage from excessive shear stress. Calculate the amount of fibrin needed based on fiber diameter and number of samples. An amount of 6 µl fibrin is used for a sugar fiber with diameter of 400 µm and length of 1cm unless stated otherwise. Calculate the number of cells needed to obtain a seeding density of 1e6 cells $cm^{-2}$. Amount of needed cells = seeding density x fiber surface

5) Withdraw 3 µl of cell-laden thrombin solution and pipette it into 3 µl fibrinogen solution. Mix them properly by pipetting up and down on ice.

CRITICAL STEP: The gel starts to crosslink directly after the two compounds come in contact with each other and it is fully crosslinked within 10 min. Therefore, it is critical that seeding takes place directly after mixing the fibrinogen and thrombin.

6) Withdraw 6 µl cell-laden fibrin solution and stamp it on sugar fiber one-by-one.

7) Repeat step 5) and 6) until the whole fiber is fully-wrapped with cell-laden fibrin.

8) Flip over the top part of the chip (seeded with cells) and assemble it with the bottom part of the chip through bolt and nut with a sealing ring in between.

6. GelMA casting and curing (Timing 5 min)

1) Prepare a 5% (w/v) GelMA solution by dissolving 500 mg GelMA in 10ml 0.5% (w/v) LAP solution at 37 °C water bath. Filter the prepared GelMA solution using 0.22 µm sterilized RC filter, and keep it at 37 °C water bath until further use.

CRITICALSTEP: Make sure to keep the lights off in the biosafety cabinet, as LAP is sensitive to light. Use blank medium to prepare 0.5% LAP solution. Wrap the prepared LAP solution and GelMA solution with aluminum foil to protect them from light. Calculate the desired volume

of GelMA solution based on the dimension of the chamber. 2 ml GelMA solution is needed for each chamber of the stainless steel chip.

2) Withdraw 2 ml of GelMA solution, and pipette it into the chamber of the chip through the top inlet to fill the whole chamber.

3) Place the 405 nm UV light on top of the chamber at a distance of 500 mm, and expose for 100 s, place the light on bottom of the chamber, and expose for 100 s.

CRITICALSTEP: 20 s illumination is needed for 200 μL GelMA solution in order to achieve complete curing.

7. Sugar dissolution (Timing 1 d)

1) Place the stainless steel chip into a petri dish, add enough medium to cover the whole sample, and incubate at 37 °C 5% $CO_2$ for overnight

8. Culture under perfusion (Timing 4 d)

1) Disassemble the silicone part from a syringe and punch a hole at center to fit with a filter. Collect tubing, luer connector, syringe, filter, silicone part with hole, and autoclave them for sterilization. Cool them down to room temperature before usage in further steps.

2) Punch a hole at the center of the silicone part of the syringe, and assemble a filter in the hole, assemble tubing with connectors, remove the piston of a syringe, fill it with fresh medium, plug the silicone part of syringe with filter into the syringe as a reservoir, connect it with tubing through connector, connect syringe for collecting perfusate with tubing through connector, and plug two connectors into the inlet and outlet of the chip respectively. Place the chip in a petri dish, place the reservoir syringe and the chip in incubator, assemble the collecting syringe on a syringe pump, and place the collecting parts outside of incubator.

3) Set perfusion mode, perfusion volume, and perfusion time. Start perfusion.

CRITICAL STEP: Calculate flow rate based on the expected shear stress and channel diameter.

9. Downstream analysis assays (Timing variable)

The constructs can be analyzed via cell skeleton staining (option A) to characterize cell morphology; and immunocytochemistry to characterize the expression of tubulin (option B).

(A) Cell skeleton staining of renal proximal tubule-on-a-chip

(i) At the desired time point, disconnect the chip from the perfusion system, take the constructs out of the stainless steel chip and transfer them to a 6-well plate containing 2 ml of PBS to wash out the medium remnants. Remove and discard the PBS from the well.

CRITICAL STEP: While pipetting, avoid touching the construct with the pipette tip in order to preserve the construct integrity.

(ii) Immerse the constructs in 3.7% formaldehyde in PBS for fixation.

!CAUTION: Formaldehyde is toxic; therefore, the fixation step should be performed entirely inside the chemical hood and the waste should be disposed of appropriately.

(iii) After 30 min, wash the construct 3x5 min with PBS.

PAUSE POINT: Fixed samples can be stored in PBS at 4 °C for a few days before further immunostaining.

(iv) Immerse the constructs in 0.5% Triton X-100 in PBS to permeabilize cell membrane.

(v) After 1 h, wash the constructs 3x5 min with PBS

CRITICAL STEP: Starting from this step, protect the samples from exposure to light to minimize photobleaching. The sample and staining solution are protected from as much light as possible by using aluminum foil and turning off the light in the biosafety cabinet.

(vi) Apply 2 drops of ActinGreenTM 488 ReadyProbesTM reagent (Invitrogen by Thermo Fisher Scientific) per 1 mL of DMEM.

(vii) After 1 hour, wash the sample with PBS for 3x5 min.

(viii) Apply 2 drops of NucBlueTM Fixed Cell Stain ReadyProbesTM reagent (DAPI special formulation, Molecular probes by Life technologiesTM) per 1 mL of DMEM.

(ix) After 30 min, wash the sample with PBS for 3x5 min.

(x) Apply Mowiol® (Sigma-Aldrich) or PBS to the sample, cover with aluminum foil, and ready for analysis or storage.

(B) immunocytochemistry of renal proximal tubule-on-a-chip

(i) At the desired time point, disconnect the chip from the perfusion system, take the constructs out of the stainless steel chip and transfer them to a 6-well plate containing 2 ml of PBS to wash out the medium remnants. Remove and discard the PBS from the well.

(ii) Fix with 4% (w/v) PFA in PBS for 30 min, and wash 3x5 min with PBS.

(iii) Permeabilize with 0.2 % Triton X-100 in PBS for 5 min, and wash twice with PBS

(iv) Block with 10% (v/v) goat serum in PBS + 0,01% Triton X-100 for 1 h.

(v) Immediately apply tubulin primary antibody (T7451) in dilution of 1:200 in blocking solution and incubate for overnight at 4 °C, and wash 3x5 min with PBS

(vi) Incubate in secondary antibody (goat-anti-mouse Alexa-conjugated ) in dilution of 1:500 in blocking solution for 1h, and wash 4x5 min with PBS.

(vii) Mount with mowiol.

(viii) Store the samples in 4 °C protected from light or directly go for analysis.

**Validation of fibrin and GelMA**

The biocompatibility of fibrin and GelMA with RPTECs is characterized, respectively.

RPTECs were seeded onto a sheet of 5% GelMA. The adhesion and growing of RPTECs on GelMA was validated. The results show that the cells were able to spread and proliferate well. The cells start spreading from day 1 onwards which can be seen by the elongated/flattened shape of the cells. A confluent cell monolayer was formed within 7 days, as displayed in **Figure S1A**.

RPTECs in the fibrin cell carrier were seeded in a wells plate. **Figure S1B** shows a clear border of the fibrin drop containing round-shaped cells, which confirms that the fibrin allows for precise placement of the cells and can hold the cells in place during seeding. Furthermore, the RPTECs were found to be able to degrade the fibrin in 1 day while starting to spread over the surface. The fibrin border disappears and cells spread and migrate over the surface. This indicates that the fibrin is degraded and not present anymore, and cells are able to free themselves. A lower cell concentration results in a longer degradation time.

**Exploration of cell seeding density**

Targeting at a specific fiber dimension (400 μm in diameter), 3 different cell densities have been compared, as shown in **Figure S2**. The cell density of 3.5e7 cells $ml^{-1}$ was validated to be the best option for this specific dimension, which will be applied as a reference in the following stages of this research.

**Exploration of sugar fiber coating realization**

In order to protect the sugar fiber from early dissolution during cell seeding and the hydrogel casting process, a hydrophobic protective coating is demanded. Considering the structure of the template to be coated, we selected dip coating as an option for applying the coating. Dip coating is a technique in which the substrate is dipped in the coating solution, after which it is retracted with controlled speed. This technique allows the full coating of complex substrates with different surface curvatures, corners, etc. For dip coating, the thickness of the coating layer depends on many factors, including the geometry of the substrate, the withdrawal speed, the surface tension of the coating liquid, as well as the coating viscosity and its evaporation coefficient, and it is determined by the balance between forces at the liquid-solid interface. The dip coating procedure is schematically shown in **Figure S3**. The substrate is submerged in the coating solution and withdrawn with a controlled speed (~3-5 s) under an angle of ~30 degrees. For repetitive dipping, the coating bath is covered with a glass slide to prevent evaporation while the coated substrate is left to dry for 10 min at room temperature between every dip/layer. Withdrawing can be done either by lifting the substrate out of the solution (**Figure S3A**) or by

using a glass pipette that redraws the fluid (**Figure S3B**). Both methods result in a similar coating thickness and protection, which means that the withdrawal technique can be chosen depending on the suitability of a specific sample. Method A is more suitable if the material on which the sugar is printed, cannot touch the coating solution, while method B allows for higher throughput and less waste coating material. As shown in **Figure S3**, a waiting step of 5 min can be implemented. The waiting step allows for the evaporation of chloroform which creates a film of higher concentration PDLGA. This film will wrap around the substrate during withdrawal. Attention should be paid to leaving the film intact while withdrawing the fluid underneath first.
Ethyl cellulose (EC) is an organic, biocompatible, and hydrophobic polymer, which has been frequently used for the encapsulation/coating of drugs. EC is soluble in several organic solvents (e.g. alcohols, ketones), but it does not dissolve in water. It can be degraded by enzymes, which approves for the further nutrition transportation. In a try-out experiment, Ethyl cellulose coating (15% in ethanol) was compared with the PDLGA coating (25 mg $mL^{-1}$) to investigate the homogeneity of the coatings and the protectiveness. EC was applied by submerging the sample for 3 min after which the coating solution is removed. It was a relatively viscous and sticky compound which made handling and uniform coating challenging. A protectiveness test with PBS showed that the EC coating was able to protect the sugar for 2-15 min depending on the local thickness of the coating. The more uniform PDLGA coating was able to protect for ~15 min. Therefore the PDLGA coating is considered more suitable for protecting the sugar against dissolving.
In order to achieve uniform cell attachment, a second coating layer needs to be applied in order to make the PDLGA-coated sugar surface more hydrophilic. Polyvinyl alcohol (PVA) is a hydrophilic, water-soluble, non-toxic polymer, which has been frequently used in biomedical applications. The 5% w/t PVA is applied to the PDLGA-coated sugar structures by 100 W, 45 s oxygen plasma treatment followed by submerging the samples into the solution for 10 min. However, it was shown that the PDLGA was not protective enough and the sugar fibers dissolved within 7-10 min. Even quickly removing access liquid from the fibers did not help. Therefore, the use of PVA as second coating material was ruled out.

**The PDLGA coating: requirements and effect**

The aim and requirements:

The aim of applying the PDLGA coating is to adequately protect the sugar template against premature dissolution during cell pre-wrapping and hydrogel casting, while not affecting the geometrical precision of the channel and the following cell culture. Ideally, the PDLGA coating

should be as thin and uniform as possible in order to minimize the difference between the designed and obtained channel dimension/ geometry, and for the coating to be degraded as fast as possible during cell culturing. However, the prerequisite is that it can provide sufficient protection for the sacrificial template, to ensure the template's stability of shape during the second coating, cell seeding, and channel formation processes, and to endow the template with enough mechanical strength and stability for the cell stamping process.

In addition to the thickness, the protecting performance of the PDLGA coating is also dependent on whether the coating is uniformly and densely distributed. As a result, the optimal selection of coating solution concentration and coating technique is crucial for realizing sufficient protection and small thickness at the same time. On the other hand, the optimal coating layer is degraded overnight just after sacrificing the fiber template, to not block nutrients from reaching cells during further cell culturing.

The effect:

We have tested different PDLGA coating solution concentrations and coating application techniques, and we characterized the resulting coating thickness, protecting performance, and cell culture compatibility. The thickness is measured through the difference of fiber diameter between after and before coating, as shown in **Figure S**4. Through the characterization of effective template protecting time, the formed channel morphology, and the following cell culture, we established the thickness of approximately 10 μm as the optimal one.

**Exploration of cell pre-wrapping seeding technique**

Regarding the first issue, the carrier in which the cells are present during seeding is crucial for the seeding quality. In case the cells are simply suspended in cell medium which is pipetted onto the sugar fiber, most cells are flushed away by hydrogel prepolymer solution during the further casting step, since cells in medium do not readily adhere to the fiber surface at their initial seeding location. To avoid this, a waiting step of at least 30 min after cell loading can be introduced, during which the medium evaporates completely, leaving only cells on the sugar fiber. However, the majority of cells do not survive this process. The first issue is solved by using fibrin as a cell carrier to hold cells in place during the subsequent lumen formation process (hydrogel casting and curing, as well as sugar dissolution) based on its fast transition from liquid to gel once fibrinogen is mixed with thrombin.

The second challenge is to load the cell-laden carrier on the sugar fiber using a pipette. Before cell loading, the fibers are double coated with Poly (DL-lactide-co-glycolide) (PDLGA) and Pluronic® F-127 (F-127). The PDLGA coating is needed to protect the fiber against premature

dissolution, but it is hydrophobic so that fibrin and cells do not readily spread on it. Therefore, F-127 is needed as a second, hydrophilic coating, but it is more hydrophobic than a pipette tip, and therefore the fluid tends to adhere to the pipette tip rather than the fiber, as shown in **Figure S5A**. After dragging the fibrin droplet back and forth for several times over the sugar fiber using the pipette, barely any fluid is left on the sugar fiber, resulting in very limited cell adherence. In an attempt to solve this issue, we proposed and tested a submerging method, illustrated in **Figure S5B**. In this case, a relatively large amount of cell-laden suspension is inserted in a specially designed container with a groove structure that matches the sugar fiber dimension. However, as shown in **Figure S5B (ii, iii)**, only very few cells adhere to the fiber, preferentially attaching to the container walls. To load a sufficient number of cells on the sugar fiber, we tested an alternative technique in which drops of cell-laden fibrin pre-polymer suspension are placed next to each other on the sugar fiber one-by-one through pipetting, shown in **Figure S5C**. However, due to the large contact angle of the cell carrier fluid on PDLGA-coated sugar fiber surface, closely placed droplets easily merge into a bigger droplet. As a result, a limited coverage of the fiber area can be achieved. In addition, the remaining big droplets (1500 μm in diameter and 400 μm in height) lead to the presence of cell volumes outside of the lumen during subsequent culturing, as shown in **Figure S5C (iv-vii)**. To improve the droplet spreading over the sugar fiber, we applied an additional coating of Pluronic® F-127 (F-127). **Figure S5D** shows that this second coating indeed decreases the contact angle of a fibrin droplet significantly, and improves droplet adhesion. The droplets on the double-coated fiber are more elongated and wrap around the whole fiber. However, the drops (1500 μm in diameter and 200 μm in height) still cause non-even coverage of the cell-laden fibrin on the fiber surface, hindering the formation of a confluent and uniform cell monolayer at the end, as shown in **Figure S5D (iv-viii)**. We explored various approaches to pre-wrapping cells around the printed sugar fiber, and finally we selected the stamping-based cell pre-wrapping seeding technique as the best option for the formation of a confluent cell monolayer.

**Calculation of the cell density in fibrin**

For a specific fiber, its surface area is calculated based on its diameter and length, and the number of drops is decided based on the fiber length, while the size of the drops is pre-calibrated targeting a specific cell suspension density and composition based on the exploration of minimal extrusion amount and the measurement of the size of the corresponding drops sitting on fiber. Accordingly, the total volume of fibrin for one fiber is calculated. Targeting a specific density of cells distributed on the whole surface, the total number of cells needed for one fiber

is calculated. Finally, the cell density in fibrin is calculated based on the cell number and the fibrin volume. This tells us how much fibrin and how many cells we need in order to prepare the cell-laden ink for one single fiber.

For example, in the case of a fiber with a diameter of $50\mu m$ and a length of *15mm*, we know that we need 2.1e6 cells and $6\mu L$ fibrin through pre-calculation and calibration. Hence, we take the corresponding amount of cell suspension after cell counting, centrifugation and discarding the supernatant. These cells are then suspended in thrombin solution, and mixed further with fibrinogen before stamping operations. To be specific, we need to prepare $3\mu L$ cell-laden thrombin solution at a density of 7e8 cells $ml^{-1}$ and then mix it with $3\mu L$ fibrinogen for covering one sugar fiber with such dimensions.

**Epithelialization process of lumen inside hydrogel**

We recorded the proliferation and spreading of RPTECs inside the lumen seeded in the fibrin cell carrier, as well as the epithelialization process of the channel over 7 days of culture. **Figure S6A and S6B** show microscopy images of RPTECs in lumens with a diameter of 400 µm and 200 µm, respectively after 1, 4, 7 days of culture. The cells on the inner wall of the lumens are observed to attach well, start spreading, and gradually cover the lumen surface. The cell coverage area increases with culture time until a uniform and dense epithelial monolayer is formed. After 7 days of culture, the epithelium could be visualized even by the naked eye, covering the entire length of the 15 mm long channel.

**Cytotoxicity of involved materials**

To evaluate the toxicity of the multiple materials involved in the whole workflow of creating the luminal model, we added them to RPTEC in standard culture after 24 h and characterized the proliferation after 48 h of culture, including the sugar material, the PDLGA coating material, and the F-127 coating solution. Control groups were cultured in basic full medium without adding any additional materials. The results shown in **Figure S7** suggest that all materials involved in our fabrication method induce only low cell toxicity, since all groups show good and similar cell proliferation.

**Display of individual channels for merged fluorescence images**

The individual channels of F-actin and Nuclei are displayed in **Figure S8**.

The structure of the stainless steel chip for dynamic culture and sugar frame structure for static culture are displayed in **Figure S9**. The individual channels of Tubulin, F-actin, and Nuclei under different FSS are displayed in **Figure S10**.

The individual channels of GFP, F-actin, and Nuclei in endothelium are displayed in **Figure S11**.

**Versatility and superiority of cell pre-wrapping seeding**

In comparison with traditional microfluidic cell seeding methods, the cell pre-wrapping seeding technique displays superiority in channels with small diameter and branched structures, as shown in **Figure S12**.

**Extended application of the cell pre-wrapping seeding technique**

It is also possible to extend the methodology to cells/tissues in other states, such as 3D cell aggregates. As an initial trial to achieve such a configuration, we used GelMA as a carrier for MDA-MB-231 cells and placed this as a droplet on the sacrificial fiber template instead of using the stamping operation. In this way, we realized a 3D heterogeneous tumor tissue around a lumen, as shown in **Figure S13**. The cell carrier is not degraded fast after lumen formation, and it will act as a long-term 3D ECM microenvironment for the cells.

In addition to cell pre-wrapping around the sacrificial template for achieving 2D cell adhesion inside the inner wall of a tubular lumen, the system can also be combined with other cell seeding techniques, such as placing the template in a cell suspension and have the cells cover the fiber from solution, mixing cells in the casted hydrogel for cell embedding in the surrounding matrix, or microfluidic injection of a hydrogel containing cells within the open lumen, i.e. after removal of the sugar, for 3D cell embedding inside the tubular channel, as shown in **Figure S14**.

**Preliminary tumor-vascular coculture model**

The development and spreading of cancer relies heavily on the presence of the surrounding vasculature, and is influenced highly by the mutual interactions between the tumor tissue and the vascular network, as illustrated in **Figure S15A**. In order to study the interaction between tumor tissues and blood vessels and to emulate the tumor development process, we designed and built a preliminary vasculature-tumor coculture model as a proof of concept, based on the printing of two parallel sugar fibers, both with diameter 400 μm and at a spacing of 500 μm. For tumor cells, 3D culture conditions better mimic the in vivo microenvironment and result in more closely representing in vivo tumor cell phenotype and function than 2D conditions. Here,

we embedded human breast carcinoma cells (MDA-MB-231) cells within GelMA to simulate the 3D microenvironment. The configuration is illustrated in **Figure S15B**. The MDA-laden GelMA prepolymer solution was injected into the blank lumen that was parallel to another lumen already pre-seeded with endothelial cells using our cell pre-wrapping seeding method and cast in GelMA, and then crosslinked to form 3D tumor tissue for simulating tumor 3D microenvironment, as shown in **Figure S15Ci-Cii**. The Umbilical Vein Endothelial Cells (HUVECs) were transfected with green fluorescent protein (GFP) protein to distinguish them from the cancer cells. The cytoskeleton staining of the construct shown in **Figure S15C** displays the intended distribution of the two cell types as designed. The tumor cells were observed to grow well in a 3D state within the lumen, and the endothelial cells formed a confluent and continuous monolayer inside the other lumen. The tumor cells were observed to grow well in 3D state within the lumen, and the endothelial cells formed a confluent and continuous monolayer inside the other lumen. The individual channels of GFP, F-actin, and nuclei are displayed in **Figure S11**. In the future, we aim to apply this model in unraveling mechanisms of tumor-vasculature interactions, cancer cell invasion and intravasation (i.e. tumor cells entering the blood vessel).

**Potential applications of the workflow**

Through the creation of sacrificial templates with specifically designed structures, e.g. fiber dimensions, shapes, spacings, as well as the loading of multiple specific cell types in combination with different options of matrix materials, the proposed workflow can be adapted to engineer a wide range of meaningful biomedical models, as illustrated in Supplementary **Figure S16**. Through the introduction of endothelial cells, endothelialized lumens can be constructed, which could be applied to investigate vascular sprouting in response to angiogenic growth factors and vascular barrier function through permeability experiments. The method can also provide a foundation for tissue engineering. Through the embedding of parenchymal cells within hydrogel cast around the sugar structures, more complicated tissue models can be constructed, such as 3D heart tissue and 3D liver tissue with an inner vascular network. A 3D model of the human blood-brain barrier (BBB) can be micro-engineered within a microfluidic chip by double-layered pre-wrapping of endothelial cells and pericytes within a specific hydrogel laden with astrocytes. These models can further be analyzed while applying biomimicking shear stresses as well as relevant biochemical factors, providing a platform for probing tissue-level response to drug inputs.

**Summary and outlook**

Our analysis of the effect of printing parameters on fiber dimensions and of the possible variations of channel structures confirms the versatility of the 3D printing method. Also, we demonstrated that stamping-seeded RPTECs are able to achieve initial attachment and uniform spreading on the printed sugar fibers double-coated with PDLGA and F-127, and the cells eventually form a confluent cell monolayer on the surface of the inner walls of the created lumens in hydrogel, showing an excellent survival rate, normal proliferation rate and cell morphology, as well as responding to different fluid shear stresses by showing flow-dependent expression of the functional marker α-tubulin. The perfusion ability as well as the excellent bio-compatibility of the proposed system were also validated. We fabricated preliminary coculture models of the vasculature-tumor interaction and the vascular-renal tubule system to demonstrate the versatility of the method. Our novel cell pre-wrapping seeding technique provides an alternative strategy for microfluidic cell seeding. The complete workflow is proposed as a basic approach to the construction and study of 3D tubular-structured OOC models. We anticipate that the proposed method may pave a new way for the construction of diverse OOC models.

With the cell pre-wrapping seeding technique, we obtain direct and precise control over the cell loading location, we can place the cells initially at the area where we want them to adhere and proliferate in an on-demand defined way, which supports the fast formation of a dense cell monolayer around the fiber. In addition, cells can be placed over entire fiber templates with flexible 3D geometries, and a confluent tubular cell monolayer inside a circular cross-sectioned lumen with a curved wall can be achieved, without any unreachable areas which are inevitable in microfluidic perfusion-based cell seeding approaches. In future work, multiple specific types of cells can potentially be independently seeded along one single fiber or on different fibers in a user-defined distribution pattern and there are a wide range of matrix materials options which are compatible with the process, enabling a diversity of applications. Next to the shown examples, our method will enable to engineer other meaningful in vitro coculture models that model the interaction between different cells/tissues/organs and to control the microenvironment. Other types of cells, such as stromal cells, immune cells, and stem cells, can further be introduced into the models, potentially providing valuable insights into the interaction between different cells/tissues/organs for systematically studying basic mechanisms, and, in the future, for applications in tissue engineering, regenerative medicine, and drug development.

**Troubleshooting**

Troubleshooting advice can be found in **Table S1**.

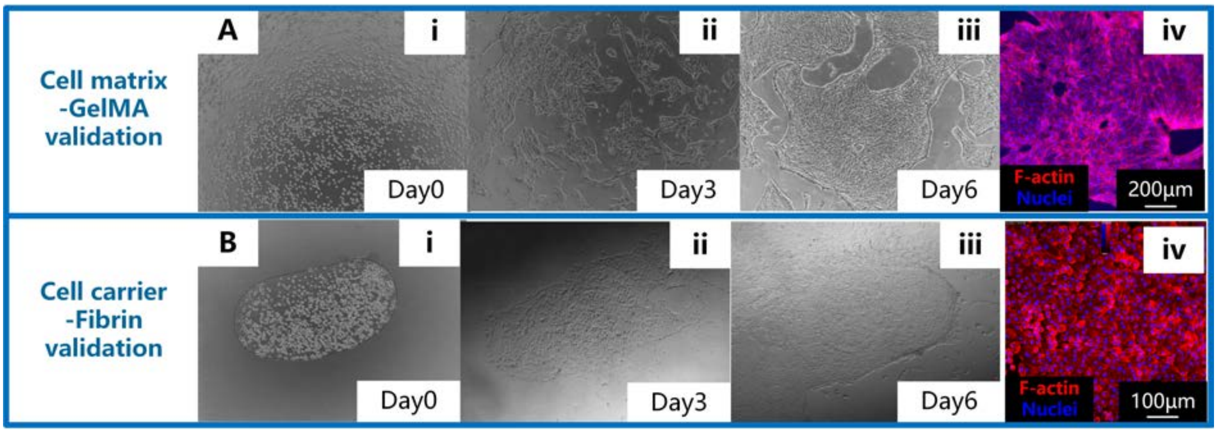


**Figure S1**. Validation of GelMA and fibrin. (Ai-Aiii) Microscopy images of RPTECs growing on GelMA after 0, 3, and 6 days of culture. (Aiv) Fluorescence micrograph of RPTECs on GelMA after 7 days of culture. (Bi-Biii) Microscopy images of RPTECs encapsulated in fibrin growing on well plate after 0, 3, and 6 days of culture. (Biv) Fluorescence micrograph of RPTECs encapsulated in fibrin on well plate after 7 days of culture. F-actin: red, nuclei: blue.

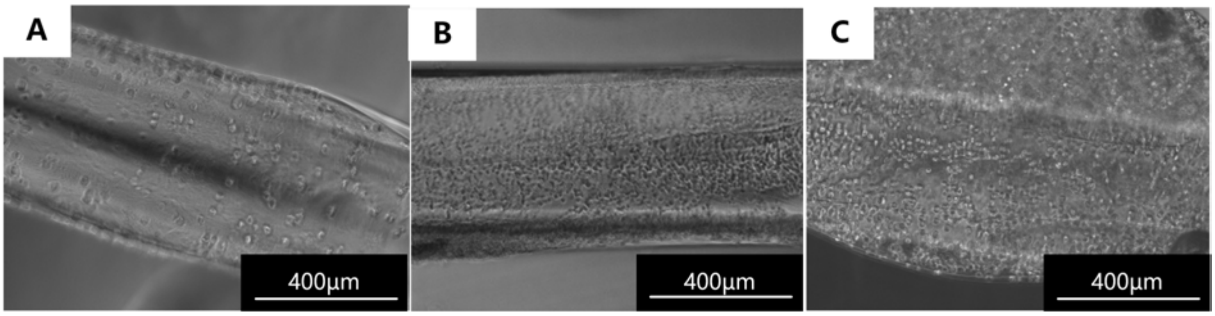


**Figure S2**. Comparison of different cell concentrations for pre-wrapping seeding. Microscopy images of sugar fibers with a diameter of 400 µm seeded with RPTECs-laden fibrin with a cell density of (A) 1e7 cells $ml^{-1}$, (B) 3.5e7 cells $ml^{-1}$, and (C) 5e7 cells $ml^{-1}$.

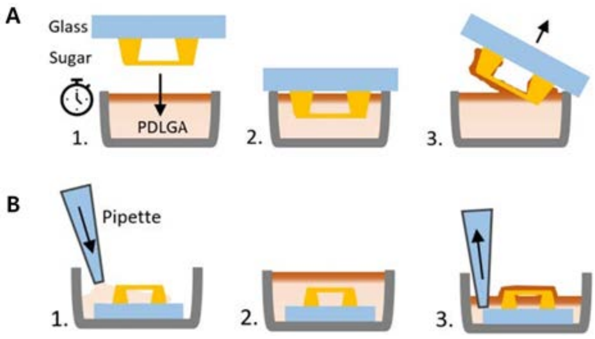

**Figure S3**. Illustration showing the coating processes of sugar-printed structures on a glass slide. (A) Starting with a waiting step of 5 min leads to chloroform evaporation and the formation of a film on top of the coating solution. The sugar structure is lowered into the PDLGA coating solution and removed after several seconds. (B) The sugar structure is placed into a glass dish and the PDLGA coating solution is added. After 5 min of waiting, the coating solution is removed by a pipette, while the film wraps around the structure.

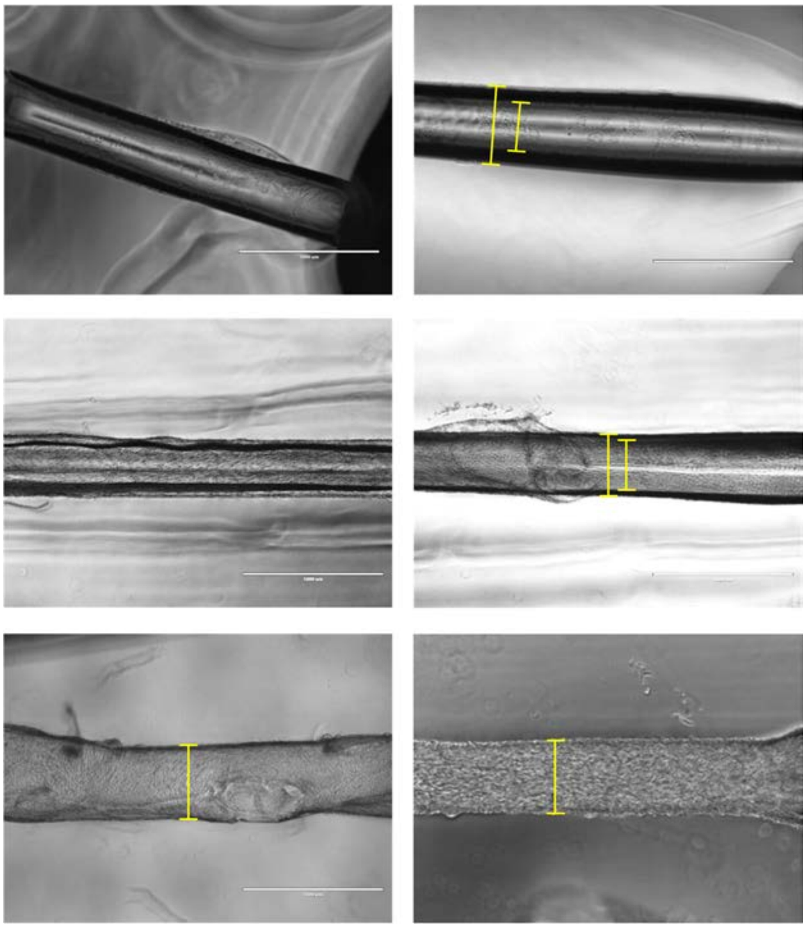

**Figure S4.** PDLGA coating thickness measurements, carried out for varying coating methods and conditions.

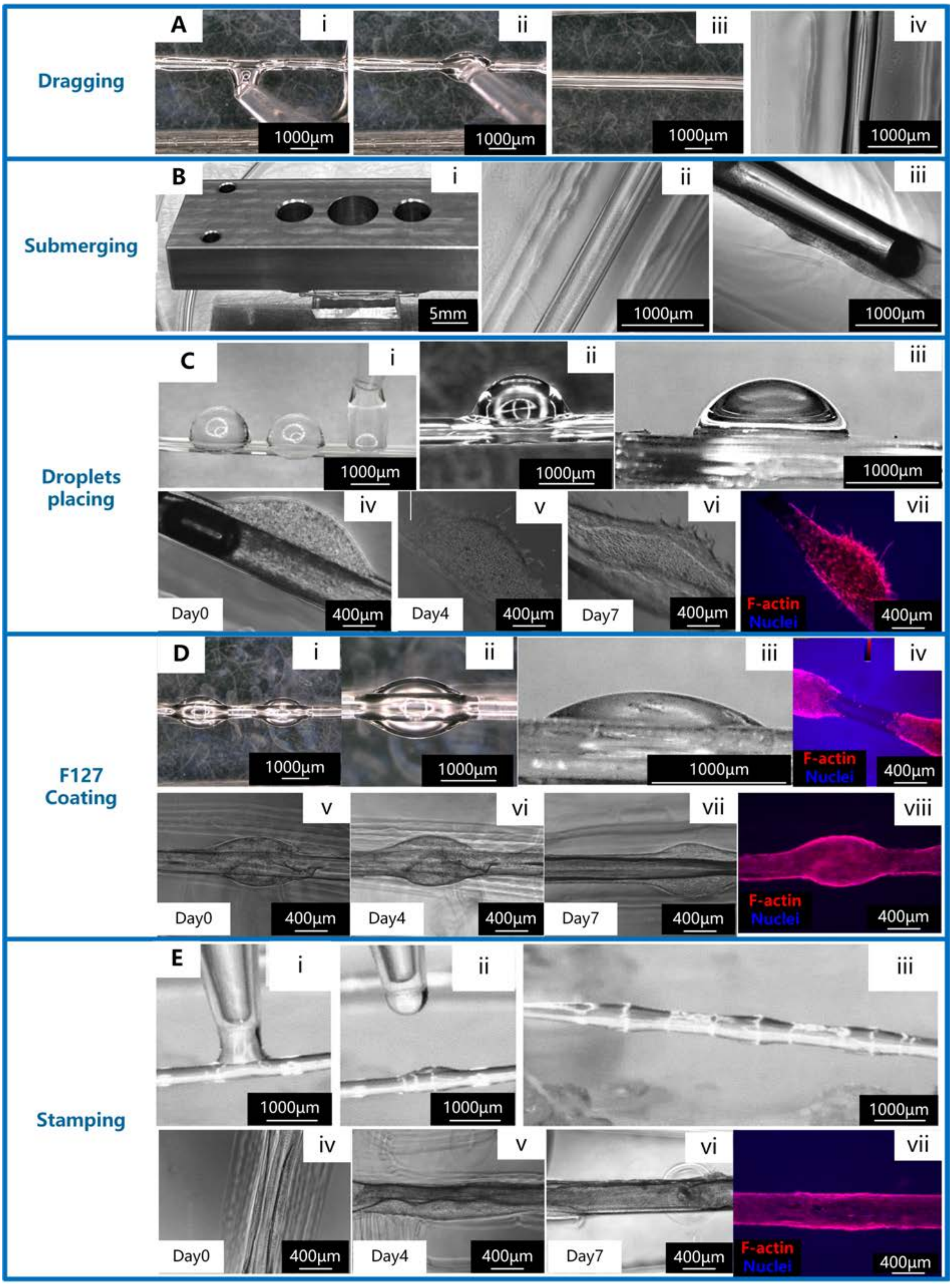


**Figure S5**. Exploration and validation of various cell pre-wrapping seeding approaches. (A) Dragging seeding technique. (Ai-Aii) Display of the dragging seeding process (tested with PBS). (Aiii-Aiv) Microscopic display of the dragging seeding result. (B) Submerging seeding technique. (Bi) Display of the submerging seeding process. (Bii-Biii) Microscopic display of

the submerging seeding result. (C) Droplets placing seeding technique. (Ci-Cii) Microscopic display of the droplets placed on PDLGA coated sugar fiber (tested with PBS). (Ciii) Microscopic display of the contact angle of the PDLGA coated surface. (Civ-Cvi) Photographs of the renal tubule epithelium in lumens with a diameter of 400 μm after 0, 4, and 7 days of culture. (Cvii) Fluorescence micrograph of F-actin/nuclei markers of the RPTEC monolayer formed by droplets placing seeding. (D) F-127 coating-based droplets placing seeding technique. (Di-Dii) Microscopic display of the droplets placed on F-127 coated sugar fiber (tested with PBS). (Diii) Microscopic display of the contact angle of the F-127 coated surface. (Div) Fluorescence micrograph of F-actin/nuclei markers of the RPTEC monolayer showing an interval between individual droplets. (Dv-Dvii) Photographs of the renal tubule epithelium in lumens with a diameter of 400 μm after 0, 4, and 7 days of culture. (Dviii) Fluorescence micrograph of F-actin/nuclei markers of the RPTEC monolayer formed based on F-127 coating. (E) Stamping seeding technique. (Ei-Eii) Microscopic display of the stamping seeding process (tested with PBS). (Eiii) Microscopic display of the stamping seeding result (tested with PBS). (Eiv-Evi) Photographs of the renal tubule epithelium in lumens with a diameter of 400 μm after 0, 4, and 7 days of culture. (Evii) Fluorescence micrograph of F-actin/nuclei markers of the RPTEC monolayer formed by stamping seeding. Based on the results, the stamping-based cell pre-wrapping seeding technique was selected as the best option for realizing a uniform confluent cell monolayer within a lumen.

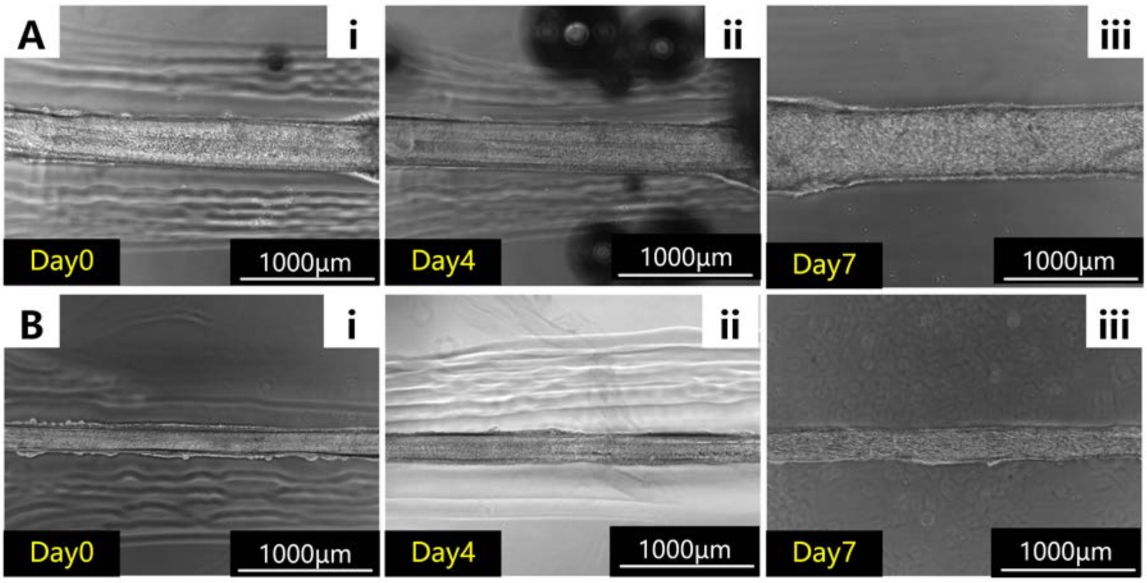


**Figure S6**. Formation of renal proximal tubule epithelium. Microscopy brightfield images of the epithelium inside lumens with a diameter of 400 μm (A) and 200 μm (B) after 0, 4, and 7 days of culture. The cells are RPTECs and the matrix is GelMA.

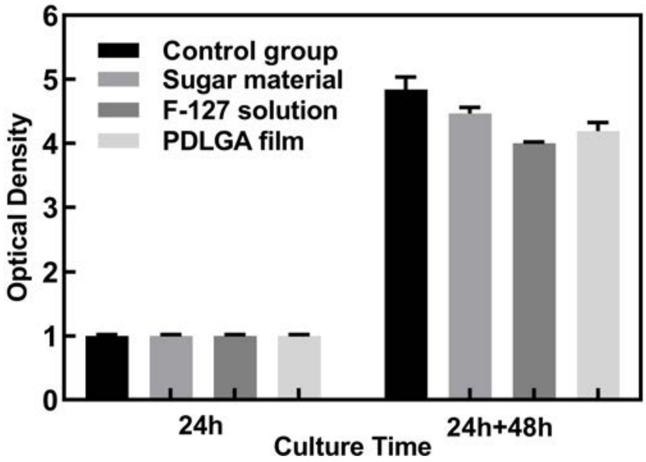


**Figure S7**. Cytotoxicity characterized by cell proliferation analysis after adding materials (sugar, F-127 solution, PDLGA film) for 48h to cells cultured at time 24 hrs.

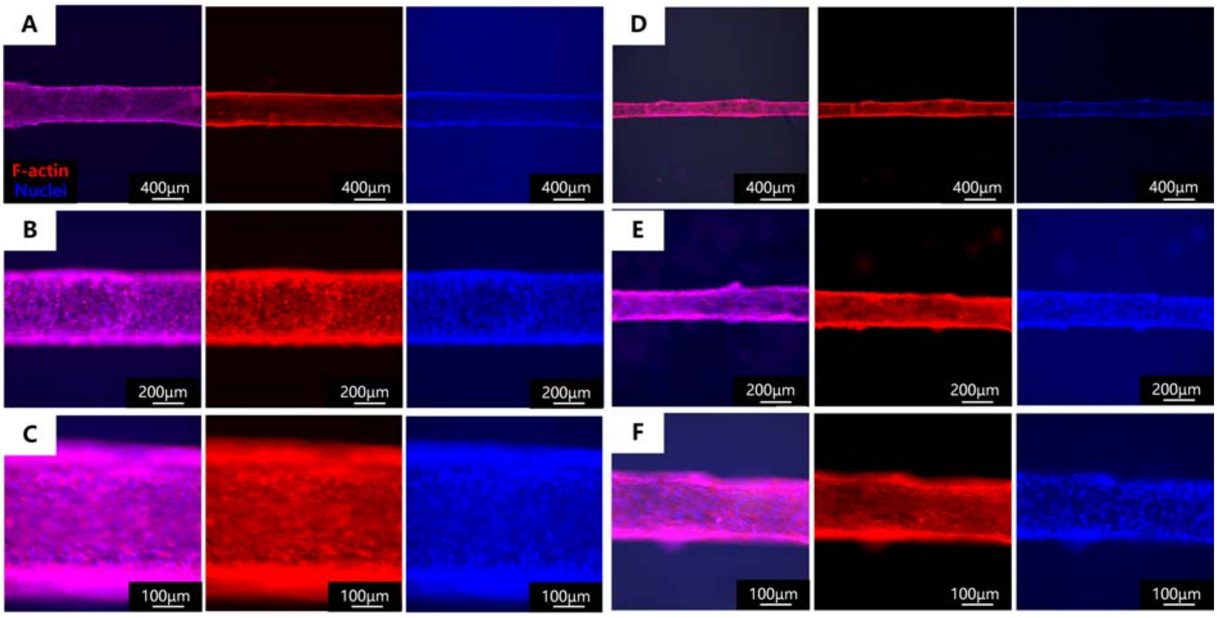


**Figure S8**. Display of individual channels of renal proximal tubule-on-a-chip stained with F-actin (red) and nuclei (blue). (A-C) Renal proximal tubule epithelium in lumens with a diameter of 400 μm. (D-F) Renal proximal tubule epithelium in lumens with a diameter of 200 μm.

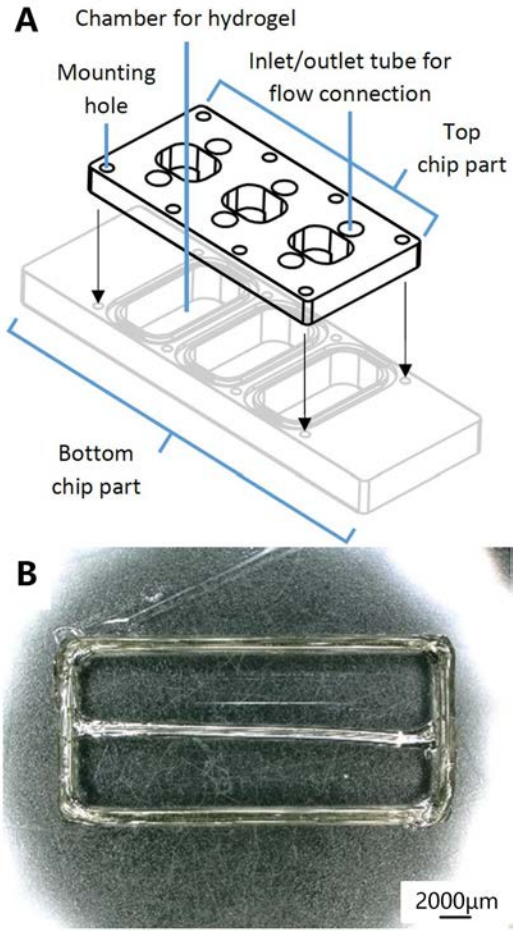


**Figure S9**. Display of structure of the (A) stainless steel chip for dynamic culture and (B) sugar frame structure for static culture.

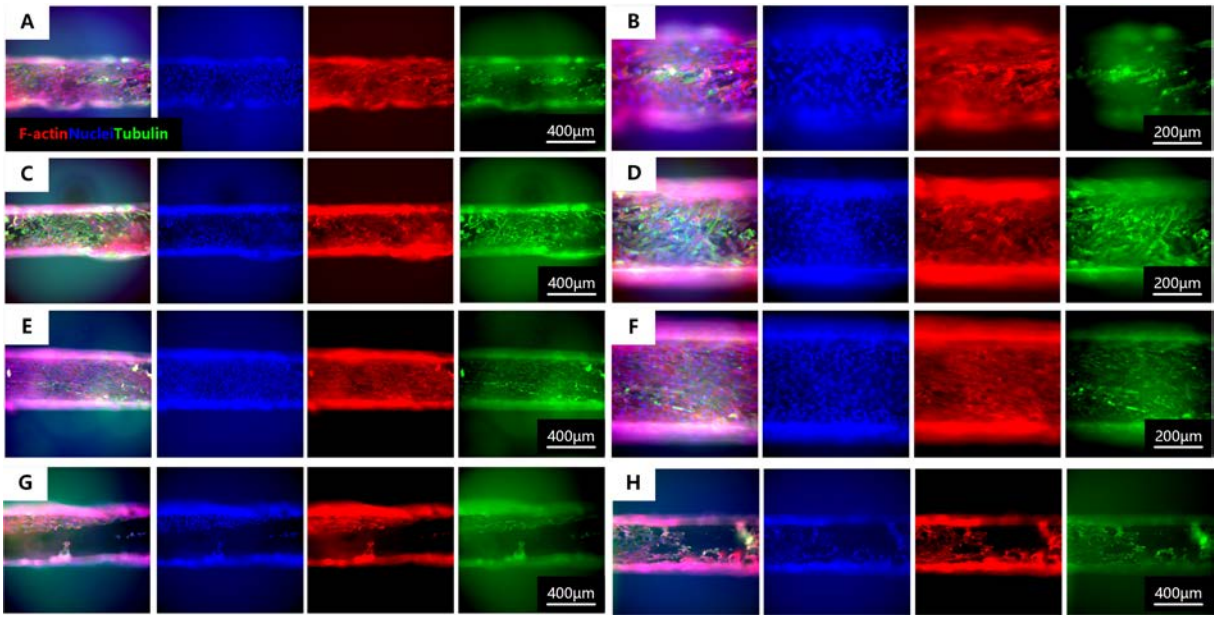

**Figure S10**. Display of individual channels of renal proximal tubule-on-a-chip under shear stress of (A-B) 0, (C-D) 0.13, (E-F) 0.26, and (G-H) 0.52 dyne $cm^{-2}$ stained with tubulin (green), F-actin (red), and nuclei (blue).

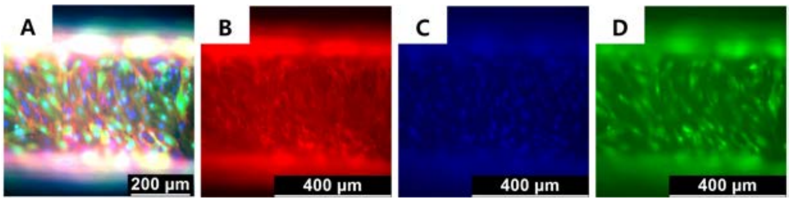


**Figure S11**. Display of individual channels of interaction models stained with F-actin (red) and nuclei (blue), HUVEC labeled with GFP (green). (A-B) Tumor-vasculature model. (C-D) Vascular-renal tubule interaction model. (E) Endothelialized lumen. (F) Lumen filled with 3D tumor tissue.

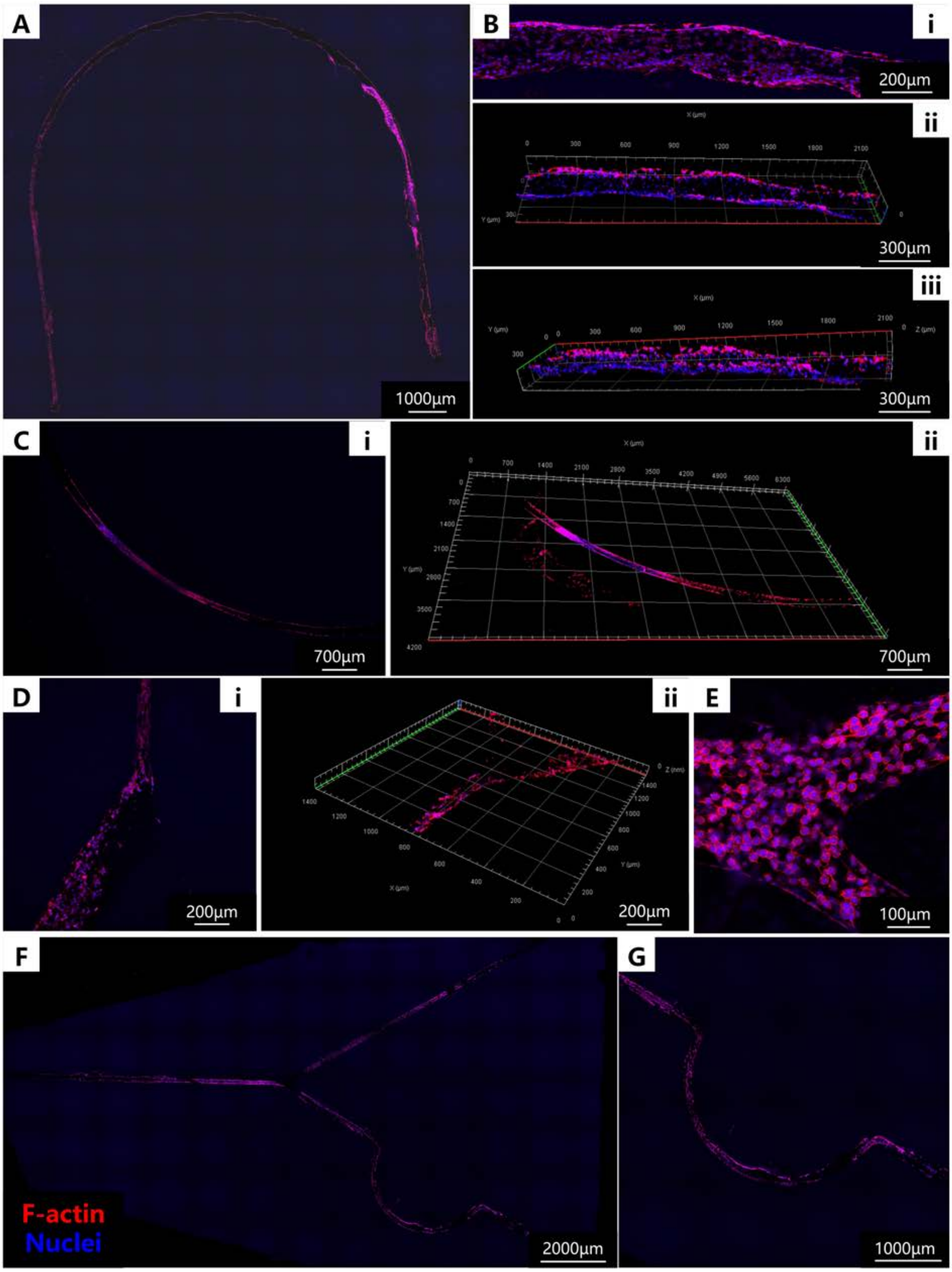


**Figure S12**. Display of superiority of cell pre-wrapping seeding over injection cell seeding. (A-C) Fluorescence micrographs of the renal proximal tubule epithelium inside curved lumens. (D) Fluorescence micrographs of the renal proximal tubule epithelium inside lumens with a branching structure seeded by conventional microfluidic injection. (E) Fluorescence micrographs of the renal proximal tubule epithelium inside lumens with a branching structure

seeded by the cell pre-wrapping technique. (F-G) Fluorescence micrographs of the renal proximal tubule epithelium inside lumens with a branching and curving structure.

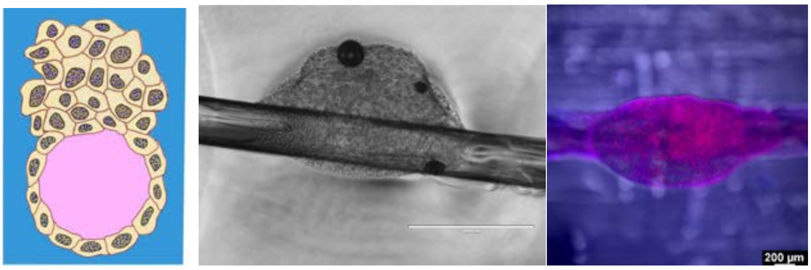


**Figure S13.** Display of 3D tumor tissue situated along a lumen established through the cell pre-wrapping technique.

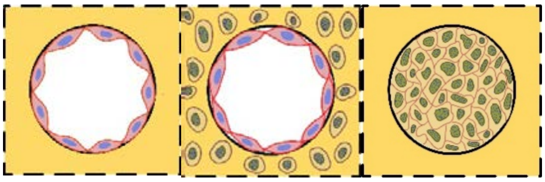

**Figure S14.** Schematic of different achievable cell distributions and states.

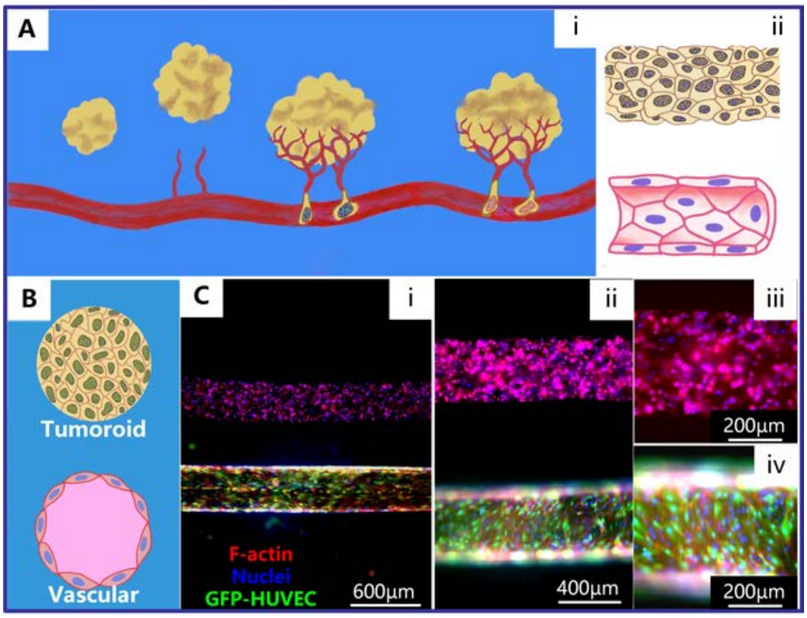


**Figure S15**. Demonstration of preliminary tumor-vasculature interaction models constructed by the cell pre-wrapping seeding technique. (A) Schematic of a tumor-vasculature interaction model. (B) Schematic diagram of the cross section of the tumor-vasculature model. (C) Fluorescent images of the engineered tumor-vasculature model at different magnifications; HUVEC transfected with GFP: green, F-actin: red, nuclei: blue.

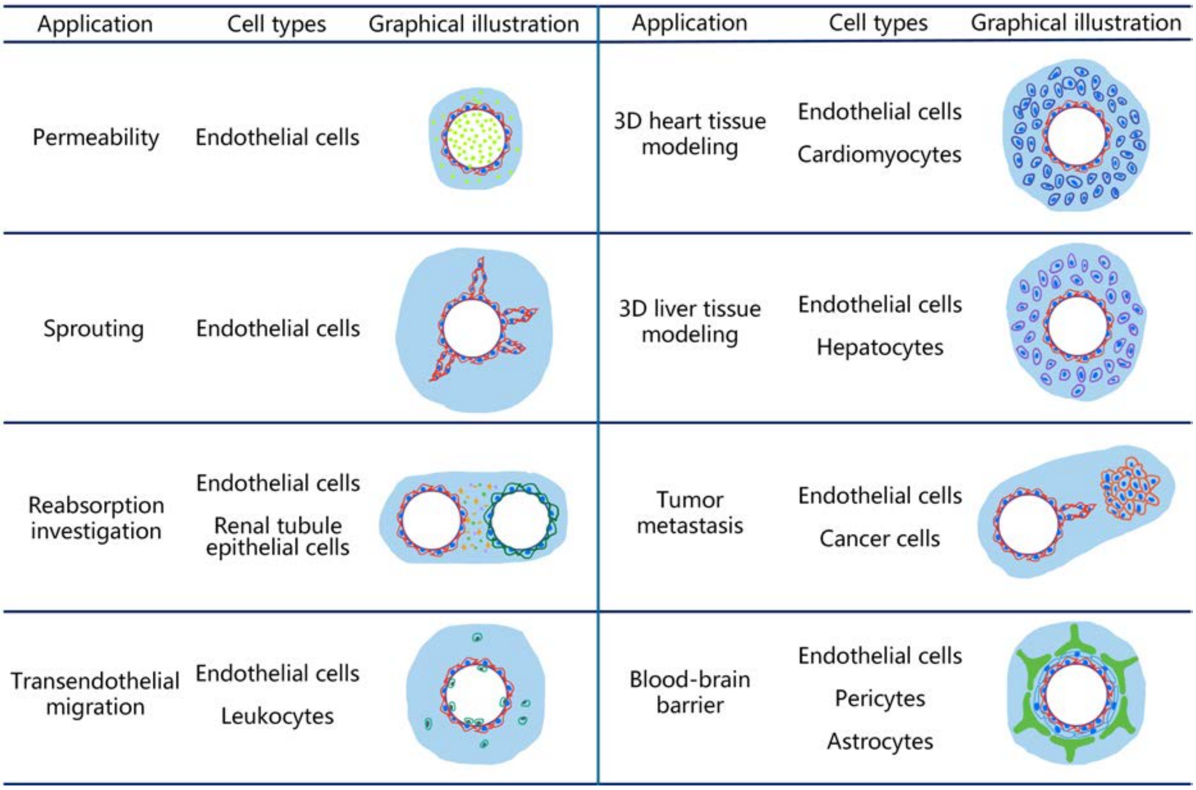


**Figure S16**. Summary of potential applications of the cell pre-wrapping seeding technique, combined with 3D sacrificial sugar fiber printing.

**Table S1**. Troubleshooting table.

| Problem | Possible reason | Solution |
|---|---|---|
| Fiber collapse<br>Fiber bend | Insufficient cooking time for sugar material preparation<br>Sugar material out of date<br>Bed temperature too high<br>Insufficient cooling will lead to sagging of the structures and loss of print geometry | Stop cooking after all water has evaporated<br>Switch on fan when printing freestanding structure |
| Geometry deformation | Not proper design of printing path<br>Insufficient cooling | Modify G-code<br>Switch on fan for real-time cooling |
| Weak point at the beginning of sugar fiber | Printing direction vertical to fan blowing for uniform cooling along the whole fiber | Printing pathway design<br>Z direction initialization |
| Sugar early dissolution | Insufficient PDLGA concentration or evaporation time<br>PDLGA film removed | Increase PDLGA concentration<br>Control coating time<br>Pay attention to the lifting operation for not removing the film |
| Uneven coating of PDLGA | Unstable lifting operation<br>Nonuniform chloroform evaporation | Replace operation by hand with automatic setup<br>Add coating solution slightly higher than fiber, so that after evaporation, the liquid surface will be lower than fiber, and coating film will uniformly wrap around the fiber automatically |
| Droplets merge | Hydrophobic surface | Apply F-127 coating |
| F-127 dissolves sugar | Soaking time too long in F-127 solution | Decrease F-127 coating time |
| Cells flushed away | Fibrin not cured | Freshly prepare fibrinogen and thrombin |
| GelMA degraded<br>Not well cured<br>Not well shaped | Low concentration<br>Insufficient UV intensity or exposure time | Increase GelMA concentration<br>Increase UV intensity<br>Increase UV exposure time |
| Cells do not spread or proliferate | Low cell density<br>Fibrin not degraded | Explore for suitable seeding density according to specific fiber dimension |

| | | |
|---|---|---|
| | Insufficient initial cell coverage in channel | |
| Fail to form a dense cell monolayer along the channel<br>Insufficient cell coverage in channel | An intact cell coverage cannot be reached (limited hydrophilicity of coated sugar surface)<br>Adhesion between cells and sugar surface is weak<br>Low cell confluency<br>Insufficient initial cell attachment at the seeding stage | Increase the initial cell number<br>Increase cultivation time<br>Multiple batch stamping |
| Low viability | UV intensity too high<br>UV exposing time too long<br>Long waiting time for fibrin curing<br>Cells dry | Control UV curing time and light intensity<br>Start timing as long as mixing fibrinogen and thrombin<br>Take into account of the chip assembly time |
| Unstable viability between different experimental groups | Changes in experimental condition and parameters | Fully-charge UV light before every single experiment<br>Freshly prepare fibrinogen, thrombin, GelMA every time<br>Control UV exposure time and distance |
| Failure to establish flow | Blockage of channel due to leakage of hydrogels from matrix space into the channels<br>Local early dissolution of sugar<br>Weak connection point between sugar fiber and sugar pillars | Adjust sugar printing parameters to achieve uniform sugar diameter<br>Uniform PDLGA coating<br>Modify chip design |
| Failure to form cell monolayer in Channel with small diameter | Lower amount of cell-laden fibrin adheres to thinner fiber due to smaller surface area in contacting with fibrin | Increase cell density for smaller channel diameter<br>(400 μm-3.5e7 cells $ml^{-1}$,<br>200 μm-1e8 cells $ml^{-1}$) |
